# A Comparative Study of Bio-Inspired Odour Source Localisation Strategies from the State-Action Perspective

**DOI:** 10.3390/s19102231

**Published:** 2019-05-14

**Authors:** João Macedo, Lino Marques, Ernesto Costa

**Affiliations:** 1 Institute of Systems and Robotics, University of Coimbra, 3030-290 Coimbra, Portugal; lino@isr.uc.pt; 2 Centre for Informatics and Systems of the University of Coimbra, 3030-290 Coimbra, Portugal; ernesto@dei.uc.pt

**Keywords:** odour source localisation, mobile robot olfaction, bio-inspired strategies

## Abstract

Locating odour sources with robots is an interesting problem with many important real-world applications. In the past years, the robotics community has adapted several bio-inspired strategies to search for odour sources in a variety of environments. This work studies and compares some of the most common strategies from a behavioural perspective with the aim of knowing: (1) how different are the behaviours exhibited by the strategies for the same perceptual state; and (2) which are the most consensual actions for each perceptual state in each environment. The first step of this analysis consists of clustering the perceptual states, and building histograms of the actions taken for each cluster. In case of (1), a histogram is made for each strategy separately, whereas for (2), a single histogram containing the actions of all strategies is produced for each cluster of states. Finally, statistical hypotheses tests are used to find the statistically significant differences between the behaviours of the strategies in each state. The data used for performing this study was gathered from a purpose-built simulator which accurately simulates the real-world phenomena of odour dispersion and air flow, whilst being sufficiently fast to be employed in learning and evolutionary robotics experiments. This paper also proposes an xml-inspired structure for the generated datasets that are used to store the perceptual information of the robots over the course of the simulations. These datasets may be used in learning experiments to estimate the quality of a candidate solution or for measuring its novelty.

## 1. Introduction

Olfaction enables the detection and localisation of distant targets, even if they are silent and invisible. In nature, most organisms use this sense to locate sources of food, danger and other individuals. But, locating odour sources in realistic environments is not an easy task. The odour particles flow with the wind and spread by molecular diffusion and turbulent dispersion, creating an intermittent chemical plume, with local voids and peaks of concentration. The intermittent characteristics of the plumes hamper the ability to estimate local gradients. Moreover, in realistic environments, the wind velocity varies, making the plumes created by weak chemical sources very hard to follow. The difficulty of locating odour sources is increased with the usage of robots, due to the uncertainties inherent to their sensors and actuators, but also, due to the chemical sensors being less sensitive than the biological counterparts, not being very selective and also due to the drift of their signals. Moreover, the process of locating an odour source has three well-defined stages, each requiring a distinct behaviour [1]:

1. **Plume finding:** where the agent must explore the environment, searching for odour cues;
2. **Plume tracking:** where the agent is in contact with the odour plume, and must follow it to a region close to its source;
3. **Source declaration:** where the agent is in the vicinity of the odour source, and must pinpoint its location.

Even though there are many real-world applications for locating odour sources, the human nose is often not sensitive enough to be able to perform this task. For that reason, trained animals have been used to assist in locating various odour sources. However, this is not a good solution, as the dangerous conditions that are usually involved risk the well-being of both animals and their trainers. Moreover, the search operations may take a long time, exhausting the searchers and making them more prone to make mistakes. In order to reduce the risk to both humans and animals, the robotics community has been actively working on methods to track odour sources. Taking the ability of animals to successfully locate odour sources, many of the existing approaches are inspired by their behaviours. To this day, these strategies were only compared from a performance perspective, i.e., which strategy works better in a given environment [2,3]. This paper proposes to study the behaviours produced by several reactive strategies and compare them from a state-action perspective. This comparison aims to achieve two goals: (1) identifying the similarities and dissimilarities in the behaviours exhibited by various strategies, in each perceptual state of different environments; and (2) understanding which are the most consensual actions to perform in face of a given perceptual state of a certain environment. Moreover, in order to perform this study, a purpose-built robotic simulator is presented and the structure of a dataset is defined. The simulator accurately simulates odour dispersion and air-flow, whilst providing considerable speed-up over real time. The dataset definition serves as a stepping stone for the creation of more data by the community. That data may be used for performing more analysis, but also for learning experiments, involving the automatic creation of search strategies.

This paper has three main contributions:

1. **Development of a Robotic Simulator.** The existing robotic simulators either do not model gas dispersion and air-flow, or do so with such detail that become too slow for being used in learning or evolutionary robotics experiments. For that reason, a new robotic simulator is developed, which is sufficiently fast to be useful in Learning and Evolutionary Robotic experiments. It models the world in 2D and uses simplified kinematics models for reducing the computational complexity. The focus is on properly modelling the air flow and chemical dispersion, for which Farrell et al.’s models [4] are used. To speed-up the simulations, the chemical dispersion and air flow are modelled a priori and played back on each simulation. This simulator is also integrated with ROS [5], allowing an easier transfer of the approaches from simulation to the real robots. The result is a simulator that is able to run much faster than real-time, being appropriate to perform many different tests, as is often required in Evolutionary Robotics.
2. **Construction of a Behavioural Dataset.** A representative set containing some of the most popular bio-inspired methods from the literature is implemented and a dataset is built, composed by state-action mappings created by each strategy. This dataset can be used by experimenters to quickly train robotic controllers. The robotic controllers are typically trained with sparse reward functions [6], i.e., functions that only provide non-zero feedback on specific events (e.g., locating a target object, colliding with an obstacle, etc). These evaluation functions are typically used as they are easier to devise than dense reward functions, i.e., functions that provide meaningful feedback for each action performed by the robot. However, in environments where the interesting events seldom take place, the robots may spend most of their time without receiving feedback. Unfortunately, the difficulty in creating appropriate dense reward functions means that there is no simple way to assign a value for each state-action mapping contained in the dataset. Nevertheless, if these mappings are created solely by good strategies, there are some guarantees that they are reasonably good.
3. **Analysis of Odour Search Strategies from a State-Action Perspective.** The various search strategies implemented are compared from a behavioural perspective, in order to understand their differences, as well as which are the most consensual actions to perform in each perceptual state. This analysis is done using the state-action mappings contained in the dataset. The mappings that are common in most strategies are considered to be the most important, as they should be necessary to successfully locate odour sources. On the other hand, the mappings that are specific for each strategy are responsible for their unique behaviours. As a result, new strategies can be built with some assurances of success, by maintaining the fundamental state-action mappings and innovating in the others.

Other contributions include the proposal of the Anemotactic *E. coli* algorithm, the integration of a plume finding behaviour in the Silkworm Moth strategy, and the usage of the wind direction to estimate the direction of odour instead of computing the concentration gradient from chemical concentration measurements at multiple locations. Using this method, it is possible to perform strategies that typically rely on two chemical sensors, such as the Silkworm Moth algorithm, using only 1 chemical sensor.

The remaining of this document is organised as follows: Section 2 describes the related work, including bio-inspired strategies for odour source localisation and robotic simulators; Section 3 presents the materials and methods used in this work, including the purpose-built simulator, the proposed dataset structure, the odour source localisation strategies used and the experimental methodology followed to analyse those strategies; Section 4 validates the proposed simulator; Section 5 describes the results of the analysis conducted to the odour source localisation strategies; Section 6 summarises and discusses the experimental results found; and Section 7 presents the conclusions of this work and provides insight into further developments.

## 2. Related Work

The robotics community has proposed many strategies for locating odour sources over the past decades. Due to the ability of animals to successfully locate odour sources, many of the existing approaches are inspired by their behaviours, which are designed to work in specific conditions. One of the most important environmental conditions is the strength and stability of the air flow. In environments where the air flow is weak or non-existent, biological organisms employ chemotactic strategies, which only use information about the chemical gradient. On the other hand, in environments containing a strong air flow, animals typically take advantage of the flow information to guide their search process [7,8]. It has been hypothesised that bio-inspired strategies may not be able to succeed in the real-world, as the existing sensors and robots are less capable than their biological counterparts [9]. While it is true that the existing hardware is far more limited than animals, it has not yet been proved that bio-inspired strategies are unable to locate odour sources in realistic environments, and the sheer popularity of these strategies seems to disagree with such hypothesis.

The present section starts by describing some of the existing bio-inspired strategies for odour source localisation. Afterwords, some of the most popular robotic simulators are presented, both general purpose ones and those meant for either learning or evolutionary experiments or odour source localisation.

### 2.1. Chemotactic Strategies

This section reports some of the most popular chemotactic methods, which are designed for locating odour sources in environments deprived of a strong air flow. In such environments, the agent uses only information regarding chemical concentration to guide its search. In environments with strong winds, the turbulent effects of the air flow create intermittent odour plumes, with many voids and local peaks of concentration, that are likely to deceive a gradient-based approach. Nevertheless, Ishida et al. [10] note that, in regions close to the odour source, the chemical gradient can be informative enough to be followed and thus, approaches designed for diffusion-dominated environments may still be able to successfully locate the odour source. An ideal strategy would alternate between chemotactic and anemotactic behaviours depending on the environmental conditions and the estimated distance to the odour source. Such an approach has already been proposed [11] and is described in Section 2.2.

#### 2.1.1. *E. coli* Bacteria

The algorithm inspired by the behaviour of the *E. coli* [2] bacteria is a reactive strategy meant to track chemical sources in diffusion-dominated environments, i.e., in environments with no air flow. It owes most of its popularity is to its simplicity, as it is a biased random walk composed only of rotations and linear motions. On each time step, the agent measures the local chemical concentration and compares it to the previous odour measurement. If the current concentration is higher, the agent makes a small rotation followed by a large straight motion, continuing searching in the same approximate direction. Otherwise, it makes a probabilistically larger rotation followed by a short straight motion, directing the search to a different direction. This behaviour is presented on Algorithm 1. There, θ and Θ are, respectively, small and large rotation angles, *l* and *L* are, respectively, small and large motion displacements.

**Algorithm 1:** Pseudocode of the algorithm inspired by the behaviour of the *E. coli* bacteria [12].

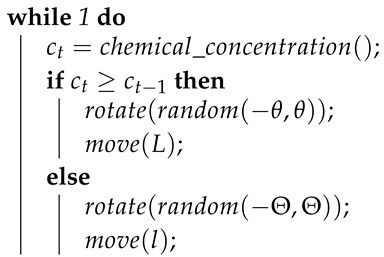



#### 2.1.2. Gradient Following

Another simple approach for locating odour sources in diffusion-dominated environments is inspired by Braitenberg’s Vehicles [13]. These strategies attempt to estimate the local chemical concentration gradient and follow it to its source. There have been many variants of this type of approaches [2], but all have some things in common. Generally, they rely on a mobile robot carrying two front-mounted chemical sensors, one at its left and another at its right. The controlling strategy typically consists of a loop, which makes the robot move forward whilst turning towards the sensor sensing the highest chemical concentration. An example of this approach is presented in Algorithm 2. There, $\left(c_{left},c_{right}\right)$, respectively, store the chemical concentrations sensed by the left and by the right sensors, whereas θ and *l* are parameters that encode the amplitude of rotation and length of motion of the robot.

**Algorithm 2:** Pseudocode of a generic gradient-following approach.

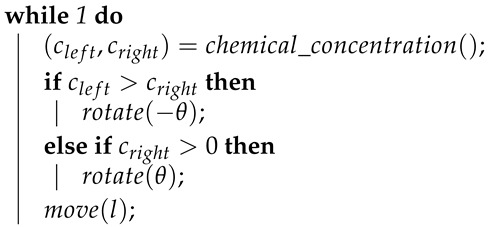



Grasso et al. [14] further developed the gradient approach and used it to control a purpose-built robot for mimicking a lobster. This robot is a two-wheeled differential-driven unit, equipped with two front-mounted chemical sensors. They proposed two control strategies. The first one, Algorithm 3, only differs from the traditional gradient approach by including a threshold of concentration, below which the robot would not turn. The second approach, Algorithm 4, adds a retreat behaviour, designed to move the robot back where it came from, whenever the chemical concentration sensed drops below a given threshold. 

**Algorithm 3:** Pseudocode of the first control strategy proposed by Grasso et al. [14].

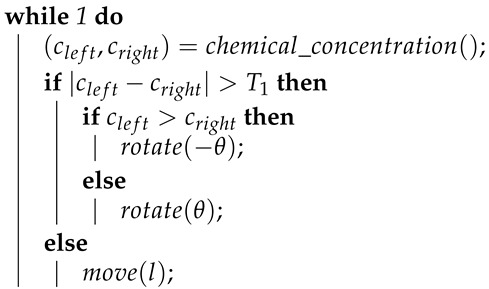



**Algorithm 4:** Pseudocode of the second control strategy proposed by Grasso et al. [14].

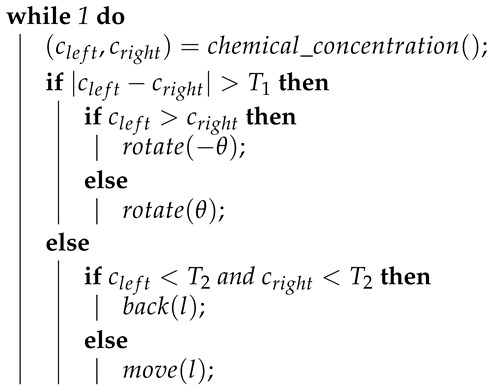



#### 2.1.3. Spiral

Spiral [15] is a chemotactic method for locating odour sources in diffusion-dominated environments. It consists of making consecutive spiral motions, restarting every time the robot considers that the gas source is closer than in the previous step. The robot estimates the distance to the source by stopping and gathering chemical concentration measurements for ΔT seconds. Those measurements are then used to compute a Proximity Index (PI), defined by Equation (1).

(1) $$PI=K_{\mu }\cdot \mu +K_{P}\cdot P\left(\Delta T_{P}\right)$$

where μ is the mean of the chemical concentration measurements, *P* is the amount of concentration peaks sensed during ΔT, i.e., local maximum over the average concentration sensed, $K_{\mu }$ and $K_{P}$ are the respective weights for the mean and sum of concentration peaks. If the newly computed PI is higher than the stored PI, $T_{PI}$, the robot considers this as a hit and restarts the spiral. Otherwise, it considers it a miss. The authors also defined a minimum threshold for the PI, $mT_{PI}$, bellow which the spiral is not restarted. If there are five consecutive misses, or three consecutive misses with PI values lower than $T_{PI}/2$, the stored PI value is lowered. Finally, the authors also devised an escape movement that is triggered when a spiral ends without any hit. In such case, the robot resets the $T_{PI}$, rotates to a random direction and moves straight for a predefined length, starting to explore a different region of the environment.

### 2.2. Anemotactic Strategies

In environments where there is a strong air-flow, animals typically employ strategies that use its direction for orienting the search, i.e., perform anemotaxis. This section describes some of the most popular anemotactic methods.

#### 2.2.1. Silkworm Moth

**Figure 1:**
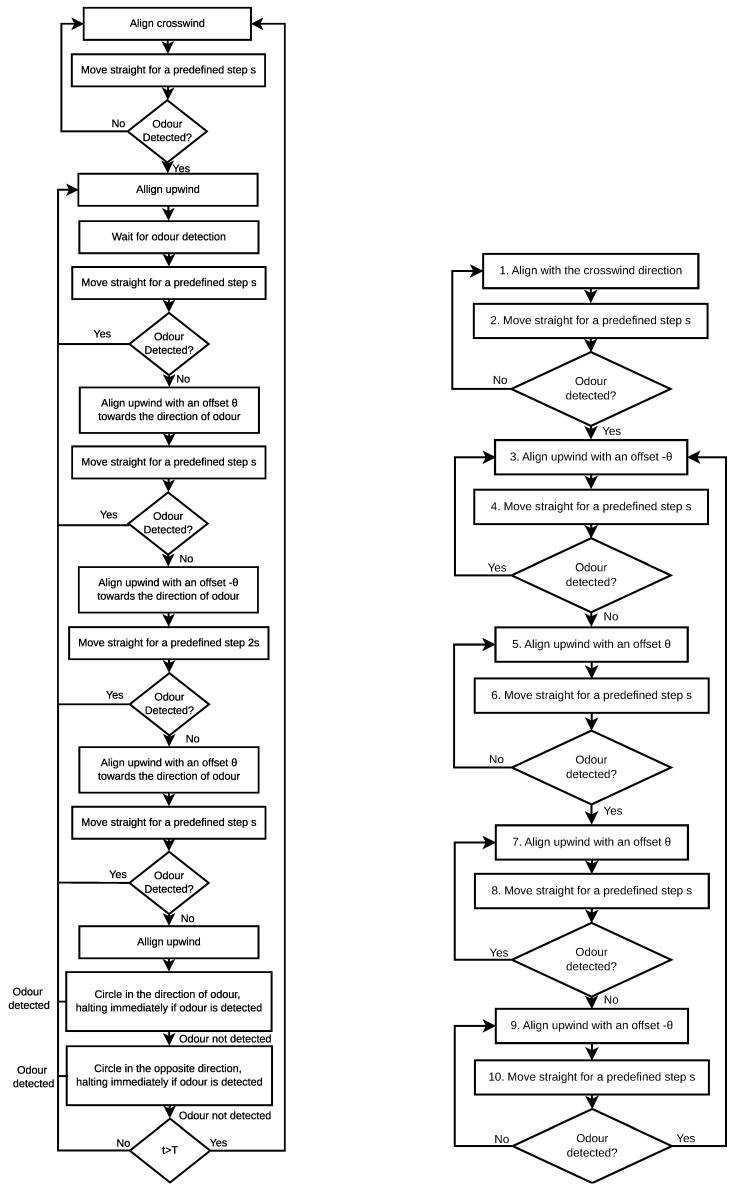
Flow charts of the modified Silkworm Moth (**left**) and Dung Beetle (**right**) algorithms, adapted from [2]. θ and *s* are user-defined parameters that, respectively, control the amplitude of the rotations and the length of the straight motions.

The Silkworm Moth algorithm is inspired by the behaviour of the Male Silkworm Moth while tracking a trail of Bombykol pheromone released by a female moth [2,8]. This algorithm assumes that the robot is equipped with two chemical sensors, mimicking the moth’s antennas. The signal from these sensors is used to compute a concentration gradient that the robot uses to select the initial direction of some of its motions. The behaviour provided by this algorithm is based on three basic movements: straight line upwind surges when detecting odour, and upwind-centred zigzag and circular motions whenever contact with the plume is lost. A flow chart of the complete behaviour inspired by the Silkworm Moth is depicted on the left of Figure 1. Liberzon et al. [16] proposed a different algorithm inspired by the behaviours exhibited by the Silkworm Moth. In their approach, the search agent uses a single binary chemical sensor and no stereoscopic information. 

The present paper modifies the standard Silkworm Moth strategy to include a plume finding behaviour based on crosswind search. This behaviour is performed at the beginning of the experiment, while the robot has not yet detected odour, and whenever it loses contact with the plume for an extended period of time. This strategy is further modified to work with a single chemical sensor, which is done by storing the upwind direction at the moment of odour detection, and use it in place of the odour concentration gradient. As the odour disperses mainly by advection, this seems like a reasonable modification.

#### 2.2.2. Dung Beetle

Similarly to the Silkworm Moth, researchers took interest in the behaviour of the Dung Beetle tracking a cow’s pat [2]. In this approach, the robot starts with a plume finding behaviour, moving crosswind in search for odour cues. Upon detecting odour, it assumes an odour-centred upwind zig-zag behaviour for tracking the plume to its source. A flow chart of this behaviour is presented on the right of Figure 1.

#### 2.2.3. Flying Insects

Harvey et al. [3] described a casting behaviour for plume finding inspired by the wasp *Cotesia rubecula*. It consists of moving back and forth across the wind, with straight motions of increasing length. In their paper, each straight motion has double the length of the previous one. The authors also compared three plume-tracking methods inspired by the behaviours of flying insects, two of which shall be used in this work:

- **Surge-Anemotaxis:** This behaviour consists of, upon detecting a chemical concentration above the predefined threshold, moving the robot upwind for a fixed length, whilst continuously readjusting its heading. If the chemical concentration sensed during the upwind surge drops below a predefined threshold, the robot will resort to the proposed casting behaviour.
- **Counter-turning:** The behaviour consists of performing an upwind zig-zag motion, while sensing odour. The angle and length of each motion is determined by the chemical concentration sensed. If the concentration is high, the robot will robot will move for a short distance and with a small offset to the upwind direction. On the other hand, when the concentration is low, the robot will move for longer distances and with larger offsets that may approximate crosswind.

#### 2.2.4. Multiphase Strategy

**Figure 2:**
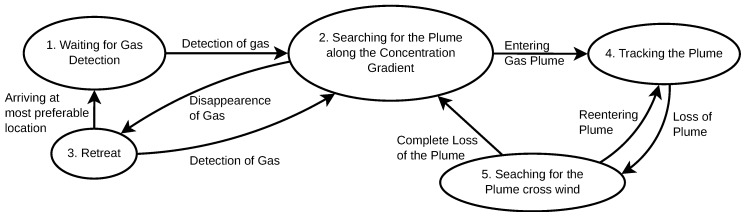
Flow chart of the Multiphase strategy proposed by Ishida et al. [11].

Real-world environments often have dynamic conditions where the wind velocity varies, being possible to exist periods with no air-flow and others with strong wind. For coping with such scenarios, Ishida et al. [11] proposed a method that combines several bio-inspired behaviours. When the robot is within the plume, this strategy employs an anemotactic behaviour inspired by the upwind surges of the Silkworm Moth. Whenever the plume is lost, the robot relies on a casting behaviour, in an attempt to re-encounter it. Casting is particularly important, as due to the random nature of odour plumes, the robot may lose contact with it. However, this anemotactic strategy was found to fail in some situations, such as whenever there are various wind sources. To tackle this problem, the authors added a chemotactic strategy which uses solely the chemical information to attempt to locate the gas source. The decision to switch between strategies is based on the chemical concentration measured. Whenever the chemical concentration is below a predefined threshold, the robot employs the chemotactic strategy. Otherwise, it will resort to the anemotactic approach. They also added a timeout condition that changes between strategies if no progress is made in a predefined time interval. A flowchart of this behaviour is depicted in Figure 2. It comprises five distinct stages:

1. **Wait for chemical detection**: The first step of this strategy consists on waiting for an initial chemical detection.
2. **Track the odour plume with the concentration gradient**: The second stage of this process consists of using the chemical concentration gradient to track the odour plume. It is employed when the gas concentration is below a predefined threshold (*odour*_*tracking*_*threshold*, set at $2.5\times 10^{6}$ molecules/cm${}^{3}$), which is considered to be caused by unstable wind conditions.
3. **Retreat:** Once the robot loses contact with the plume, it resorts to the retreat behaviour, which consists of moving back in the direction from where it came from. As soon as odour is detected, the robot moves back to stage 2, unless if in the previous step it has sensed the highest chemical concentration in its memory. In such case, it considers that the current location is the most promising for detecting chemical information, and moves to stage 1.
4. **Track the chemical plume:** If the sensed chemical concentration is higher than a threshold, the robot resorts to an upwind search behaviour for tracking the plume. During this behaviour, the concentration gradient is used to bias the motion of the robot towards the centre-line of the plume.
5. **Crosswind search for plume finding:** When the robot loses contact with the plume during the tracking phase, it will perform crosswind search. As soon as the plume is re-encountered, the robot will go back to stage 4, tracking the plume upwind. If the two crosswind directions have been searched twice and no gas has been detected, the robot will go back to stage 2.

For the purposes of the study to be conducted in the present paper, a plume finding behaviour is added to this strategy. As was previously described, the initial step of the multiphase strategy consists on remaining still, waiting for chemical detection. However if the robot is far from the plume, no detection will ever take place. For that reason, a plume finding behaviour is added to this strategy, consisting on moving crosswind until a chemical patch is detected. At such time, the robot will start performing the multiphase strategy from state 1.

### 2.3. Simulation of Olfactory Experiments

Simulators are abstractions of the real-world and for that reason, real-world experiments are of the utmost importance to validate the proposed methods. Unfortunately, real-world experiments are time consuming, sometimes impractical and potentially costly as equipment may be damaged and consumables are spent. Simulators provide an easier, faster and cheaper way to test and even though they may not provide the realism needed to properly validate the proposed methods, they are often realistic enough to assess the initial approaches. Moreover, the usage of simulators allows experimenters to create the environmental conditions in which they intend to test their approaches. There are many simulators for robotics, but in this section only those that are related with this work are addressed.

#### 2.3.1. Stage

Stage [17] is an open source 2D simulator that is currently distributed as part of the Robot Operating System framework (ROS) [5]. It was originally developed as part of the Player/Stage project [18], where it served as a background simulator for Player, a TCP/IP server for communicating with various devices. With the advent of ROS, Player lost many users, and Stage was ported into this new framework. Stage has a strong community support, developing many models of both robots and sensors. Two other advantages of Stage are its high scalability, being able to simulate many robots in real time, and high degree of fidelity, with the controllers tested in it usually transferring well to the real robots.

One of the available modules for Stage is PlumeSim [19]. This module was developed for Player/Stage and provides an easier method to integrate information regarding odour dispersion into simulated or real experiments. This software package has 3 modes of operation: (1) use a mathematical model of gas dispersion to generate the odour concentration data; (2) use a log file containing measurements of chemical concentration which may be generated artificially or collected from real-world experiments; and (3) use a commercially available software of computational fluid dynamics to generate the odour dispersion. Thus, PlumeSim enables the experimenters to simulate odour dispersion data with various degrees of accuracy. However, none of its operation methods is able to generate data that takes into account the influence of the robots’ motions in the dispersion of odour, which depending on the size of their bodies and speed of their motion may not be negligible.

#### 2.3.2. Gazebo

Gazebo [20] is another open source robotic simulator distributed freely with ROS. It is a 3D simulator that relies on third party libraries to accurately model the environment and robots. The robots’ bodies are constructed from a set of 3D primitives, such as spheres, cubes and cylinders that are joined together by different types of joints. Each primitive has different attributes such as mass and friction that enable them to behave realistically when acted upon, i.e., when pushed, pulled or knocked over. Taking in consideration the attributes of the various components, Gazebo accurately simulates the dynamics of the complete robot bodies, also performing collision detection. This simulator also benefits from a large community developing high-level robot behaviours and making them available through ROS. The main disadvantage of Gazebo is its high computational complexity, which results from modelling real-world phenomena in three dimensions.

#### 2.3.3. JBotEvolver

JBotEvolver [21] is an open source robotics simulator developed with the express intent of being a platform for evolutionary robotics (ER) experiments, having already been used in many ER studies. It is composed by two main modules, the JBotSim which is responsible for modelling the environment and robots, and JBotEvolver which is responsible for the evolutionary component. Compared to Stage and Gazebo, this simulator offers considerably fewer pre-programmed models. It focus solely on 2D simulations, featuring only a model for differential-driven robots and its evolutionary component is focused on evolving neural networks. However, its open source nature makes it possible for users to extend it, modelling other types of robots and implementing evolutionary algorithms for producing different types of controllers. The main advantage of JBotEvolver is the ability to speed up the controller evaluations, which are typically the most time consuming part of ER experiments. It does this by using 2D models, rather than 3D, but also by enabling multiple evaluations to run in parallel.

#### 2.3.4. Gaden

Gaden [22] is a realistic simulator that models odour dispersion in 3D. It supports 3D-CAD models of the environment and relies on OpenFoam, an open source computational fluid dynamics toolbox to accurately model the gas dispersion in the environment. Gaden also provides models of different gas sensors, such as metal-oxide (MOX) and photo-ionisation detector (PID). As a result, Gaden is a prime candidate to test an approach prior to real-world validation. Unfortunately, high fidelity results in increased computational costs, making such simulators slower. Gaden tackles this problem by computing the air flow and chemical dispersion offline, and simply playing back this datasets in the robotic simulations. However, the gas dispersion in the environment is stored in a 3D grid map, which, depending on the size of the cells, may require considerable memory resources.

## 3. Materials and Methods

This section briefly presents the materials and methods used for studying the odour source localisation strategies. It starts by describing the proposed dataset structure, moving on to present the considered strategies. Afterwards, the developed simulator is described and, finally, the experimental methodology to analyse the various strategies is presented.

### 3.1. Developed Simulator

The analysis made to some of the most commonly used robotic simulators, Section 2.3, revealed that they are not suited for this work. The general-purpose robotic simulators and those that are aimed for odour source localisation tasks, focus on achieving high degrees of fidelity in detriment of simulation speed. On the other hand, the faster simulators, developed for Evolutionary Robotics experiments, do not model wind flow and gas dispersion phenomena.

**Figure 3:**
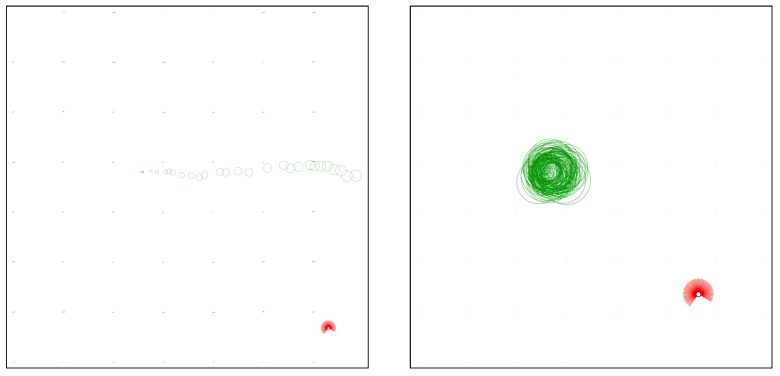
Screenshots of the developed simulator showing the advection-dominated (**left**) and diffusion-dominated (**right**) environments used in this work. Each environment is an enclosed rectangular space, containing a single odour source (thick green circle). In the advection-dominated environment, the wind flows from the left to the right of the environment, carrying the odour filaments (thin green circles). Conversely, in the diffusion-dominated environment, the weak and unstable air flow is unable to carry the odour filaments away from the source, clustering in its vicinity. The molecular dispersion of odour is represented by the increase of the diameter of the filaments. The wind velocity is computed on a grid which covers the entire arena. The wind vector of each vertex of the grid is shown as a black line, indicating its direction and speed. Each screenshot contains one mobile robots (black circle), equipped with the necessary sensors to perform odour source localisation. The red lines drawn over the robot represent the beams from its simulated Laser Range Finder, used for obstacle detection.

For those reasons, this work presents a newly developed simulator that uses the well-accepted models for odour dispersion, air flow and chemical sensors proposed by Farrell et al. [4]. A visualisation interface was built using OpenCV [23], which allows visual assessment of the world but may also be disabled for faster simulations. Two screenshots of example configurations are presented in Figure 3. Due to the usage of OpenCV, its coordinate system is employed, i.e., the origin is the top left corner, with the *x*-axis pointing to the right and the *y*-axis pointing downwards. In order to reduce the computation time, the odour and wind data are pre-generated and played back on every simulation. To guarantee that each trial starts with fully dispersed odour, the datasets are generated with double the intended duration for the trial and only their second halves are used. The resulting simulator is a good compromise between accurately modelling the real-world and execution speed, achieving a speed up of approximately 1705 times over real time. In fact, while logging the datasets, the 1200 simulation steps used in each simulation of this work only took approximately 0.352 s in a desktop computer with an Intel i7-4770K CPU, 16 GB of ram, a SSD hard drive and running Ubuntu 16.04. This speed-up makes the developed simulator adequate for learning and evolutionary robotics experiments, where the evaluations of the candidate solutions are typically the most time consuming part of the process [24]. The proposed simulator models the world as rectangular arenas, which may be empty or contain obstacles. Moreover, each environment may contain one, or several chemical sources, whose locations are drawn randomly from a specified region. The reason for randomising the location of the chemical sources is to make sure that the robots may not simply learn a route directly to them. Moreover, the start location of each robot is also drawn randomly from a pre-defined region. All of the parameters used in this simulator can be easily reconfigured to create distinct environments. Table 1 presents the parameter values used in this work, some of which are specific for each environment.

#### 3.1.1. Wind

The wind is modelled by a 2D grid of square cells, each with a width equal to 15% of the width of the whole arena. The reason behind such a coarse grid is that the model used [4] is not meant to model the small-scale turbulent phenomena of the wind, but rather its large-scale advection dynamics that have a greater impact on gas dispersion. The initial wind velocity is constant and predefined by the user. However, over the course of the simulation, the speed and direction of the wind at each vertex of the grid varies according to the following equations:

(2) $$\frac{\partial \bar{u}}{\partial t}=-\bar{u}\frac{\partial \bar{u}}{\partial x}-\bar{v}\frac{\partial \bar{u}}{\partial y}+\frac{K_{x}}{2}\cdot \frac{\partial^{2}\bar{u}}{\partial x^{2}}+\frac{K_{x}}{2}\cdot \frac{\partial^{2}\bar{u}}{\partial y^{2}}$$

(3) $$\frac{\partial \bar{v}}{\partial t}=-\bar{u}\frac{\partial \bar{v}}{\partial x}-\bar{v}\frac{\partial \bar{v}}{\partial y}+\frac{K_{x}}{2}\cdot \frac{\partial^{2}\bar{v}}{\partial x^{2}}+\frac{K_{x}}{2}\cdot \frac{\partial^{2}\bar{v}}{\partial y^{2}}$$

Zero-mean Gaussian noises are posteriorly added to the updated wind vector. The standard deviation of the noise added to the wind speed is set at 10% of its initial speed, whereas the standard variation of the noise added to its direction is specified in Table 1. The wind sensed by a robot is computed as a weighted average of the wind computed on the four grid vertexes surrounding it. The weight used for each vertex is the relative distance of the robot to it.

#### 3.1.2. Odour

In this work, the source emits odour at a constant rate of 0.2 filaments/s, equating to $8.3\times 10^{9}$ molecules/s of chemical substance. The filaments are initially released with a radius *R* of 0.03 m which increases, at each time step, according to Equation (4).

(4) $$\Delta R=\frac{\gamma }{2\cdot R}\cdot \Delta t$$

where ΔR denotes the change in the filament’s radius, γ controls the growth rate and is set at 0.01 m/s and Δt is the simulation step, which is set at 0.5 s.

Each robot senses odour through a simulated gas sensor that is governed mainly by three variables: (1) the amount of chemical substance accumulated in the sensor; (2) the decay rate; and (3) the detection threshold. The sensor’s output signal is 0 if the accumulated chemical concentration is below the detection threshold, which is set at $0.4\times 10^{6}$ molecules/cm${}^{3}$. Otherwise, it is equal to $c_{t}$, until the sensor saturates with concentration values over $5\times 10^{6}$ molecules/cm${}^{3}$. At that point, the sensor will continue to output a signal corresponding to $5\times 10^{6}$ molecules/cm${}^{3}$ of odour, until the accumulated concentration drops below this value. Equation (5) describes how the accumulated odour in the sensor *c* is updated.

(5) $$c_{t}=\alpha \cdot \left(C\right)+\left(1-\alpha \right)\cdot c_{t-1}$$

where $c_{t}$ is the accumulated odour concentration at time *t* and α is the decay rate. *C* is the instantaneous odour concentration encountered by the robot at that location, and it is computed by Equation (6).

(6) $$C=\sum_{i=1}^{N}\frac{Q}{\sqrt{8\Pi^{3}R_{i}^{3}}}\cdot \exp\left(-\frac{d_{i}^{2}}{R_{i}^{2}}\right)$$

where *Q* is the amount of chemical substance within each odour filament (i.e. $Q=\bar{Q}/n$), *N* is the total number of filaments in the environment, $R_{i}$ is the radius of filament *i* and *d* is the distance of the robot to it.

#### 3.1.3. Robots

The simulated robots are two-wheeled differential-driven units measuring 50 cm in diameter. Each robot is equipped with the necessary sensors for locating odour sources, i.e., a laser range finder (LRF) for obstacle avoidance, an anemometer and a gas sensor. The gas sensor and anemometer were already described in the previous sub-sections. The laser range finder has a maximum range of 1.5 m, and emits 50 beams equally spaced over a field of view of 1.5π radians centred on the front of the robot. On each simulation step, the robots move with a given linear and angular velocity. Effects of friction, acceleration and uncertainties of the actuators are neglected.

### 3.2. Behavioural Dataset

The behavioural dataset proposed in this paper stores for each simulation step, the perceptual state and the action taken by each robot. One of reasons for creating a behavioural dataset is to cope better with sparse reward functions [6], which are typically used in robotic experiments. These functions only provide meaningful feedback on specific events (such as colliding with an obstacle), and thus, may cause the robots to act blindly for extended time periods. Despite this disadvantage they are still often used, as they are much simpler to devise than dense functions, i.e., functions that provide non-zero feedback for every action of the robot. Smart and Kaelbling [25] attempted to minimise the issues arising from sparse reward functions by using demonstrations created by a human operator to speed up the learning process. In an initial phase of the learning process, a human tele-operated the robot in the environment, exposing it to the events of interest. Once the experimenters considered that the robot had collected sufficient information, they enabled the learned policy to control it.

Another reason for creating this dataset is the ability to compare the strategies from a behavioural perspective. In this work, the strategies are regarded as black-box controllers, and compared solely based on the state-action mappings produced.

#### 3.2.1. Structure of the Dataset

The dataset has a structure inspired by xml. The data generated on each step is enclosed between the <step s> and </step s> tags, where *s* is the index of the simulation step. Within each simulation step, the data of each robot is enclosed between the <robot r> and </robot r> tags, where *r* is the id of the robot. Even though the present work only uses a single robot, this format is already designed to be scalable for multi-robot experiments. The information stored by each robot is divided into 5 categories: odometry, odour, wind, scan and target, which encompasses the action made by the robot. A template of a complete step of data is presented in Table 2.

### 3.3. Study of Odour Source Localisation Strategies

The developed simulator is used to study the behaviours produced by the various strategies under different conditions. Two environments are created with distinct initial wind velocities and degrees of stability, as well as different arena dimensions. Moreover, for each environment, each strategy is tested in 100 independent trials, each started with a different random seed. As the position of the odour source and the initial position of the robot are chosen randomly from specified regions, they will vary from trial to trial. The parameters of the simulator used for these experiments are presented in Table 1.

Using the information contained in the behavioural dataset, the different strategies are compared from a state-action perspective. This analysis is performed for each environment separately, to gain insight about the behaviours of the various strategies in distinct environmental conditions. However, the method for performing the analysis is the same. It starts by using the data collected to construct higher-level features that better describe the state and actions of the robots, Section 3.3.1. Afterwards, the analysis is made separately for both environments, starting by clustering the states perceived by the robot and building histograms of the actions performed in each state, Section 5.1.1 and Section 5.2.1. Then, statistical hypothesis tests are used to assess the statistically significance of the differences between the behaviours displayed by the various strategies in each state, Section 5.1.2 and Section 5.2.2. Finally, the actions performed by all strategies in each state are merged and their histograms are plotted to draw conclusions about the most consensual action performed in each situation.

#### 3.3.1. Designed Features

Based on the information contained in the dataset, we hand-designed a set of features that better describe the states experienced by the robot, as well as the actions taken by it. These features are presented in Table 3.

Apart from these features, we also use the raw signal from the odour sensor (i.e., the continuous values of odour concentration sensed by the robot (SO)) to discriminate between the concentrations sensed by the robot without explicitly defining a threshold.

#### 3.3.2. Methodology for the Statistical Analysis

In order to be able to draw robust conclusions, statistical hypothesis tests are applied to the state-action mappings of each cluster of states. In each simulation trial, each strategy experienced the various states for different numbers of simulation steps. As such, it is necessary to perform a pre-processing step, creating representative datasets with the same number of samples. This is done by merging the samples collected over the different trials, obtaining a single set for each strategy and state. These sets are then truncated to have the same number of samples. In this process, the algorithms that do not have at least 30 mappings for a given cluster are discarded, thus ensuring the validity of the statistical analysis.

The next step of the analysis consists of assessing whether the data follows normal distributions, which is done by applying the Kolmogorv–Smirnov test. The *p*-values obtained will show whether, at the chosen confidence level of 95%, the mappings produced by all strategies follow normal distributions. If they do, it is possible to employ a parametric test, such as the Independent One-Way Anova, for assessing whether there are statistically significant differences in the entire set of algorithms. Otherwise, a non-parametric test must be used. The next step of the statistical analysis consists of a pairwise comparison of the various strategies, for each cluster of states where statistically significant differences were found. If all data follows normal distributions, this can be done with the Independent *t*-test. Otherwise, a non-parametric test must be applied. The Bonferroni Correction is used for adjusting the significance value.

### 3.4. Considered Strategies

In this work we shall focus on comparing the behaviours produced by a subset of the chemotactic and anemotactic strategies described in Section 2.1 and Section 2.2. These strategies are meant to work under distinct environmental conditions. Chemotactic strategies are designed to operate in environments deprived of a strong air flow and thus, only use chemical concentration measurements to guide the search process. On the other hand, anemotactic strategies are designed to operate in the presence of strong winds, using flow information to guide the search. In this work, chemotactic and anemotactic strategies shall be employed in advection-dominated and diffusion-dominated environments. It is important to note that it is not guaranteed that these strategies shall perform poorly outside of their intended environments. In fact, Ishida et al. [10] state that chemotactic strategies may still perform adequately in the presence of strong winds, as in regions close to the odour source, the chemical gradient can be informative enough to be followed. Moreover, the diffusion-dominated environment used in this work still contains a weak and highly variable flow (as many indoor environments do) and thus, it is still possible for the anemotactic strategies to perceive the direction of the wind. The set of strategies considered in this work is composed by:

1. *E. coli* chemotaxis (EC), Section 2.1.1;
2. Grasso et al.’s first strategy (G1), Section 2.1.2;
3. Grasso et al.’s second strategy (G2), Section 2.1.2;
4. Spiral (SP), Section 2.1.3;
5. Modified Silkworm Moth (SM), Section 2.2.1;
6. Dung Beetle (DB), Section 2.2.2;
7. Casting and Surge-Anemotaxis (SA), Section 2.2.3;
8. Casting and Counter-turning, (CT), Section 2.2.3;
9. Ishida et al.’s Multiphase strategy (MP), Section 2.2.4;

The parameters used by the various strategies, as well as their respective values, are listed in Table 4. Moreover, we propose an adaptation of the *E. coli* algorithm for advection-dominated environments, which is presented in Section 3.4.1.

#### 3.4.1. Anemotactic *E. coli* Bacteria Algorithm

The original *E. coli* bacteria algorithm was devised for acting in environments similar to those of the natural organism, i.e., diffusion-dominated environments. However, in advection-dominated environments, animals typically take advantage of the flow information to guide their search processes. As a result, an adaptation of the traditional *E. coli* algorithm is proposed to better cope with advection-dominated environments. In this new version, whenever the robot senses a higher chemical concentration, it will rotate upwind with an added Gaussian noise and make a long straight motion. On the other occasions it will rotate crosswind with a Gaussian noise and then make a short straight motion. This method, hereon referred to as *Anemotactic E. coli* strategy (AE) is presented on Algorithm 5. All of the parameters are the same as those used in the original *E. coli* algorithm, presented in Table 4.

**Algorithm 5:** Pseudocode of the Anemotactic *E. coli* algorithm.

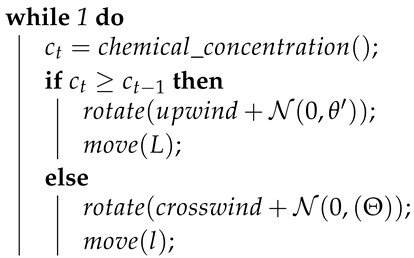



## 4. Simulator Validation

**Figure 4:**
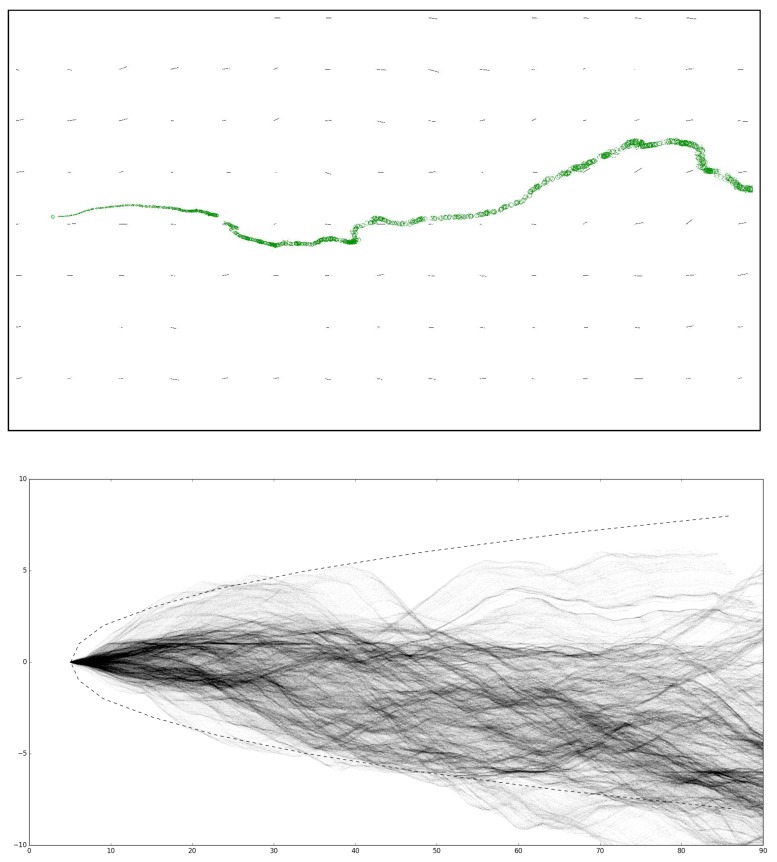
(**Top**) A 100 m by 50 m cut-out of the meandering plume generated by the proposed simulator. (**Bottom**) Time-averaged chemical concentration emitted over 600 s (faded curves, whose transparency is proportional to the mean concentration), surrounded by the Gaussian Plume model (dashed lines). The chemical plumes and Gaussian Plume model were generated using the same parameters as Farrell et al. [4].

This section presents a validation of the proposed simulator, comparing its results to the simulated and real-world data provided by Farrell et al. [4]. In order to do this, the parameters of the simulator are modified to match those used in [4]. These parameters are only used for validation purposes and are presented in Table 5. The first step of the validation process consists in visually assessing the shape and behaviour of the created plume. The top plot of Figure 4 presents a cut-out of the generated plume, measuring approximately 100 m alongside the wind direction and 50 m crosswind. The plume depicted exhibits the expected meandering effect as well as the turbulent diffusion, matching the results obtained in [4].

The following step of the validation process consists of simulating the meandering chemical plume for 10 min and plotting its time-averaged concentration. The bottom plot of Figure 4 presents the time-averaged chemical concentration as curves of different transparencies, proportional to the amount of concentration present at each location. That plot also presents the boundaries of the Gaussian Plume model (dashed curves), which models the average chemical concentration emitted by a source for a long time period. As can be seen, the odour filaments dispersed stay mostly within the boundaries of the Gaussian Plume model. The discrepancies visible at over 50 m downwind from the source may be due to the choice of parameters or, most likely, due to 10 min not being enough simulation time for accurately reproducing a Gaussian Plume model.

**Figure 5:**
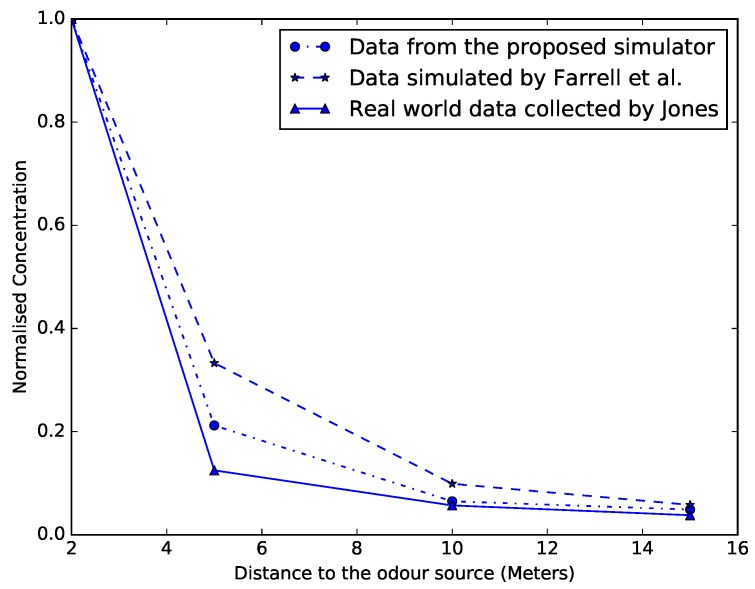
Plot of the chemical concentrations sensed at 2, 5, 10 and 15 m downwind from the source, averaged over 600 s. Each dataset is normalised by the data collected at 2 m downwind from the source.

The third step of this validation consists of collecting chemical concentration measurements at various distances downwind from the source, and comparing them with the simulated data generated by Farrell et al. [4] as well as to the real-world data collected by Jones [26]. This data was used by Farrell et al. to validate their models and thus, it is appropriate for validating the proposed simulator. Figure 5 plots the time-averaged chemical concentrations collected by the three methods over the course of 600 s. The chemical concentration is measured at four locations, respectively, 2, 5, 10 and 15 m downwind from the source. Due to the different magnitudes in the data collected by Jones and Farrell et al., in [4] the mean concentrations at each point were normalised by the values obtained at two meters from the source. The same approach is applied to the data generated by the proposed simulator, enabling a direct comparison. As can be seen from Figure 5, the proposed simulator not only exhibits an equivalent behaviour to that of Farrell et al., but it even provides better approximations to the real-world data collected by Jones. This phenomenon may be due to the choice of some parameters that are not explicit in [4] (e.g., Kx), but also due to the different distributions used for modelling turbulence and diffusion.

Table 6 presents the remaining statistics used by Farrell et al. to validate their models. Similarly to [4], the skewness and kurtosis are expressed in non-dimensional form. Analyzing the table, it is possible to see that the results of the simulator proposed in this paper are quite similar to those of Jones and Farrell et al., even without knowing all the parameters that Farrell et al. used in their validation. The skewness and kurtosis of the plume generated by the proposed simulator are typically lower than those of the plume simulated by Farrell et al., meaning that in the case of the proposed simulator, the chemical concentrations measured at those locations are more varied and less biased. However the skewness and kurtosis values of the plume generated by the proposed simulator behave similarly to those of Farrell et al.’s plume, increasing as the distance to the chemical source increases and becoming more similar to the target values. The intermittency (i.e., the percentage of time spent not detecting odour) of the chemical plume generated by the proposed simulator also increases as the distance to the chemical source increases. This value is quite similar to that obtained by Farrell et al. at 2 m from the chemical source. At the other two locations (i.e., 5 and 10 m downwind from the source) the intermittency of the plume produced by the proposed simulator is higher than that of Farrell et al., approximating better the real-world data collected by Jones. The peak to mean ratio (i.e., the ratio between the peak and mean concentration values) computed for the proposed simulator also behaves similar to the results of Jones and Farrell et al., increasing as the distance to the source increases. Finally, the standard deviations of the chemical concentrations measured at each location are analysed. Due to the magnitudes of the concentrations measured by Jones and Farrell et al. being quite different, the standard deviations computed for each location are divided by the corresponding mean concentrations. The ratios computed from the data of the proposed simulator behave similarly to those computed from the data of Farrell et al., increasing as the distance to the source increases. Moreover, the ratios of the proposed simulator at 2 m and 5 m from the chemical source are between the values obtained by Farrell et al. and Jones.

## 5. Results

This section presents the results obtained in the two experimental environments: (1) the advection-dominated and the (2) diffusion-dominated. For each environment, the perceptual states are clustered and, for each cluster, histograms of actions are created. The actions made by the robots are characterised by the DCTU feature (Section 3.3.1) but, for improved readability, they are simply referred to as moving downwind, crosswind or upwind. The next step of the analysis consists of using statistical hypothesis tests to validate which of the studied strategies behave significantly different in each perceptual state. Finally, for each environment, the state-action mappings of all strategies are merged and used to study which are the most consensual actions to perform in each perceptual state.

### 5.1. Environment 1: Advection-Dominated

The section reports the analysis made on the data gathered from Environment 1. Recall that this environment is a large square arena (70 × 70 m) with no obstacles, a single odour source and a strong, relatively stable, air flow.

#### 5.1.1. State Clustering

**Figure 6:**
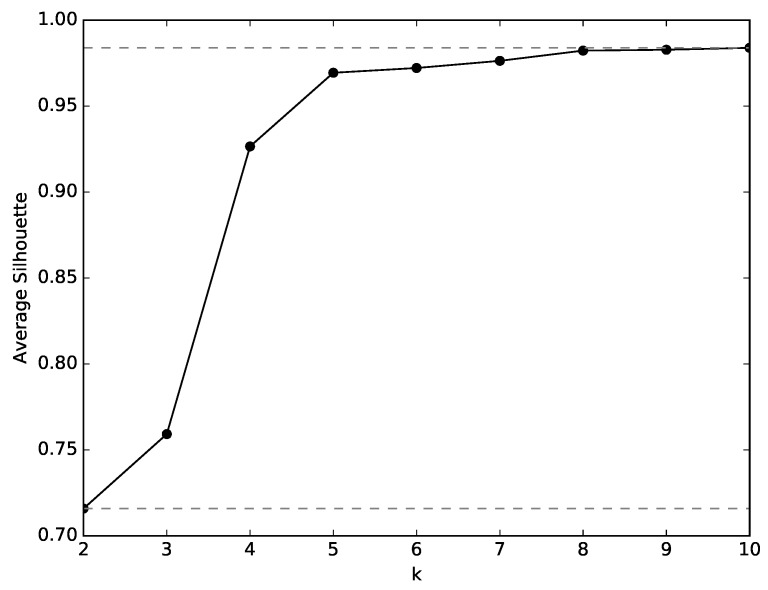
Average silhouette values for *k* between 2 and 10, applied to the results of Environment 1.

As described in Section 3.3, the first stage of the analysis consists of finding a meaningful set of clusters of states that define the experiences of the robot. The clusters are created by the k-means algorithm and the silhouette method is applied for selecting the appropriate value of *k*, i.e., the adequate number of clusters. *k* values between 2 and 10 were tested and the corresponding silhouette values are plotted in Figure 6. As can be seen, the silhouette values show the largest increase between 2 and 5. The gains past k=5 are not worth the increased complexity, and as such, this value is selected for *k*.

**Figure 7:**
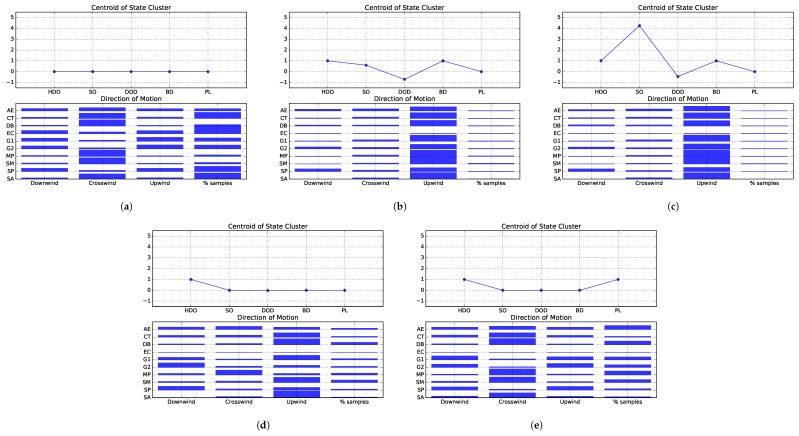
5 state-action mappings for environment 1. The top of each sub-figure presents the centroid of the respective state cluster. The bottom of each sub-figure presents the stacked histograms of relative frequency of the actions taken by each strategy in the respective state. A fourth bar is added to the histogram of each strategy, representing the percentage of simulation steps spent perceiving the corresponding state. (**a**) State 1: the robot has yet to detect any odour concentration; (**b**) State 2: the robot is sensing low concentrations of odour; (**c**) State 3: the robot is detecting high concentrations of odour; (**d**) State 4: the robot has recently lost contact with the chemical plume; (**e**) State 5: the robot has not detected odour for an extended time period.

The second step is the construction of histograms of relative frequency of the actions performed in each state. Those actions are represented by the DCTU feature, Section 3.3.1, which computes the cosine of the robot’s goal vector in relation to the upwind direction. It then divides the range of the cosine values into 3 equal intervals, and yields −1, 0 or 1 if the robot’s motion is more aligned with the downwind, crosswind or upwind direction. The centroids of the states clusters and corresponding histograms are plotted in Figure 7a–e. Those figures also contain an extra bar, indicating the relative amount of simulation steps spent by each strategy perceiving the respective state.

The first state cluster, from hereon referred to as State 1, represents the plume finding stage of the odour source localisation process. In this state, the robot has not yet sensed odour, and must explore the environment searching for chemical cues. Analyzing Figure 7a it is possible to see that the surge-anemotaxis (SA), silkworm moth (SM), dung beetle (DB), counter-turning (CT) and multiphase (MP) approaches prefer to move in the crosswind direction. Conversely, the other approaches favour moving in the upwind and downwind directions. The exception is the anemotactic *E. coli* algorithm (AE), which does not exhibit a clear preference of motion. The second state represents situations where the robot is detecting odour, yet at a relatively low concentration. Analyzing the histogram plotted in Figure 7b, it is visible that most strategies favour moving upwind in this situation. The exceptions are the EC, which never perceived this state, and spiral (SP), which due to its spiraling trajectories, gives similar preference to both the upwind and downwind directions. State 3, plotted in Figure 7c, corresponds to situations where the robot is sensing a high odour concentration. Similarly to the previous state, in this scenario, most strategies prefer moving in the upwind direction. The exceptions are SP, which prefers moving mostly in the downwind and upwind directions and EC, which never perceived this state. The fourth state, Figure 7d, corresponds to situations where the robot has recently lost contact with the plume. In this state, most strategies prefer to move upwind. Conversely, the AE does not exhibit a clear preference, while Grasso et al.’s second strategy (G2) and SP favour moving downwind. Moreover, the MP gives preference to moving crosswind and the EC never perceived this state. Finally, state 5, depicted on Figure 7e, encompasses situations where the robot has detected odour in the past, but has lost contact with the plume for an extended time period and thus, should employ a plume finding behaviour. In this scenario, SP and the strategies proposed by Grasso et al. give preference to moving in the upwind and downwind directions. The other strategies favour moving crosswind. The exceptions are EC, which never perceived this state and its anemotactic variant, which does not exhibit a clear preference.

From Figure 7a–e it is also possible to see that all strategies spent relatively small amounts of time sensing odour (states 2 and 3). The majority of time was spent in the plume finding stage (state 1) and attempting to re-encounter the plume after it had been lost (states 4 and 5). Here too, its visible that the strategies tend to spend more time in state 5 than in state 4. This is somewhat to be expected, as a robot perceiving state 4 is more likely to re-encounter the chemical plume and move to state 2. Otherwise, it may continue not sensing odour and evetually transition to state 5.

#### 5.1.2. Statistical Analysis

Having visually assessed the differences between the state-action mappings of the various strategies, this section focuses on assessing the statistically significant differences between their behaviours. This comparison is done by applying statistical hypothesis tests to the state-action mappings of each cluster of states. In each simulation trial, each strategy experienced the various states for different numbers of simulation steps. As such, a preprocessing step was performed, as described in Section 3.3.2. In this process, the *E. coli* algorithm was discarded from the statistical analysis, as it had only perceived the first state, i.e., it never encountered odour. At the end of this process, there is one dataset per strategy and state cluster. The datasets used for each cluster have all the same length, which ranges between 22,040 samples (state 1) and 483 samples (state 3).

The next step of the analysis consists of assessing whether the data follows normal distributions, which is done by applying the Kolmogorv-Smirnov test. The *p*-values obtained show that, at the chosen confidence level of 95%, the mappings produced by all strategies follow normal distributions. Thus, it is possible to employ a parametric test, such as the Independent One-Way Anova, for assessing whether there are significant differences in the entire set of algorithms. The results of the Independent One-Way Anova show that there are statistically significant differences, as all *p*-values are either equal to 0 or several orders of magnitude lower than the significance value. As a result, the next step of the statistical analysis consists of a pairwise comparison of the various strategies, for each cluster of states.

The pairwise comparison is done with the Independent *t*-test, which results are presented in Table 7. The Bonferroni Correction is used for adjusting the significance value. As there are 36 comparisons per state, and this test yields a two-tailed *p*-value, the original significance value is reduced to approximately 0.00069.

This test shows that, in state 1, i.e., in the plume finding stage, there are 23 statistically significant differences between the behaviours of the various strategies. The most consensual strategy is SP, whereas the most disruptive behaviours are exhibited by G1, G2 and SA. State 2 corresponds to the situation where the robot is sensing a relatively low odour concentration. In this state, there are a total of 31 statistically significant differences. The most dissimilar strategies are SP, MP and SM. In the third state, when the robot is detecting high odour concentrations, there are a total of 29 statistically significant differences. The most disruptive behaviours are exhibited by the SP, being significantly different from all other strategies. The most consensual strategy is DB, being significantly different from 3 other strategies. The fourth state corresponds to situations where the robot has recently lost contact with the plume. In this state there are 35 statistically significant differences between the behaviours of the various strategies. The only strategies which do not behave significantly different from each other are AE and MP. Finally, in the fifth state, when the robot has lost contact with the plume for an extended period of time, there are 29 statistically significant differences out of the 36 comparisons made. The most dissimilar strategies are G1, G2 and SP each being significantly different from all other strategies. Conversely, the most consensual behaviours are exhibited by DB, SA and CT, each being significantly different from the five strategies.

### 5.2. Environment 2: Diffusion-Dominated

The behavioural analysis of the strategies is repeated for the second environment. This environment consists of a square arena with a quarter of the dimension of the first one. It also differs in the air flow, and consequently in the odour dispersion. While the first environment has a strong and relatively stable air flow, here the air flow is very weak, and highly variant. This leads to the odour remaining in the vicinity of the source, as it is common in diffusion-dominated environments.

#### 5.2.1. State Clustering

**Figure 8:**
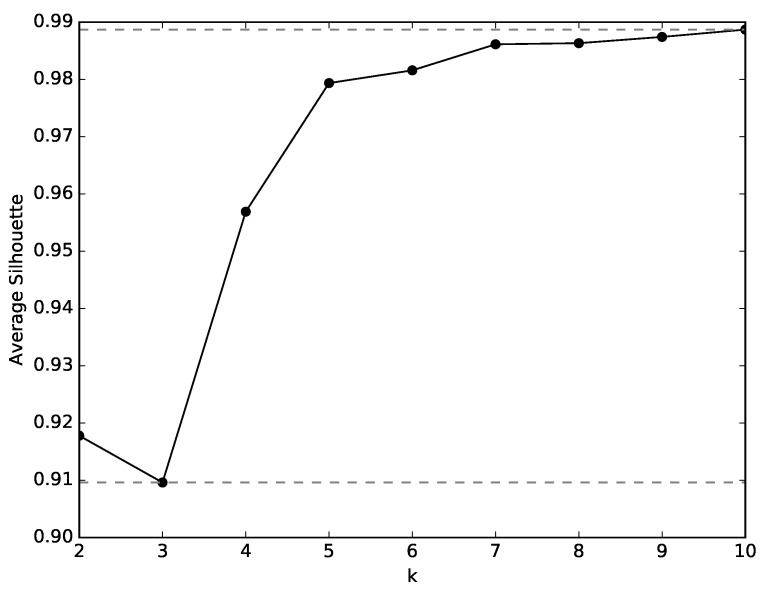
Average silhouette values for *k* values between 2 and 10, applied to the results of Environment 2.

Similarly to the analysis made in Environment 1, the states experienced by the several robots are clustered and, for each cluster, the histograms of relative frequency of each action are constructed. The adequate number of states is found through the silhouette method, which results are plotted in Figure 8. There, it is visible that the silhouette values decrease from k=2 to k=3, and greatly increase from k=3 to k=5. However, from k=5 onwards, the silhouette value increase slowly. For that reason and for a more compatible analysis with that of environment 1, k=5 is selected.

**Figure 9:**
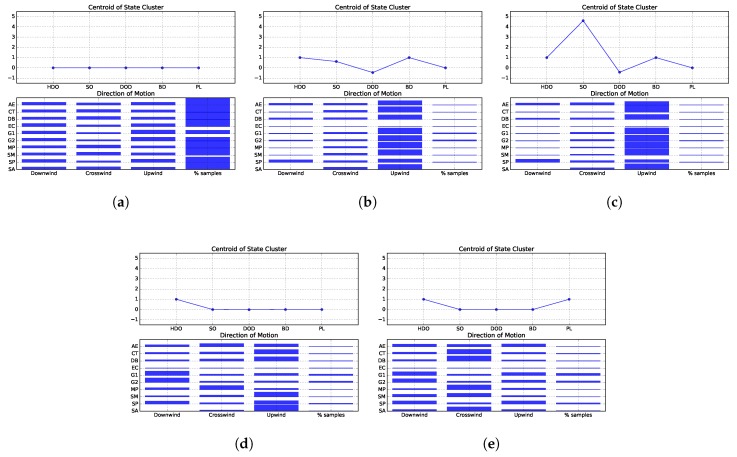
5 state-action mappings for environment 2. The top of each sub-figure presents the centroid of the respective state cluster. The bottom of each sub-figure presents the stacked histograms of relative frequency of the actions taken by each strategy in the respective state. A fourth bar is added to the histogram of each strategy, representing the percentage of simulation steps spent perceiving the corresponding state. (**a**) State 1: the robot has yet to sense any odour concentration; (**b**) State 2: the robot is detecting low concentrations of odour; (**c**) State 3: the robot is detecting high concentrations of odour; (**d**) State 4: the robot has recently stopped detecting odour; (**e**) State 5: the robot has lost the plume for an extended time period.

The first state, depicted in Figure 9a represents the plume finding stage, where the robot has yet to sense odour and must explore the environment, searching for the chemical plume. The histograms for this state do not show clear preferences in the direction of motion. Nevertheless, it seems that the strategies proposed by Grasso et al., spiral and the *E. coli* algorithm slightly favour moving in the upwind and downwind directions, rather than crosswind. Figure 9b presents the results obtained for state 2. This state represents situations where the robot senses reasonable low amounts of chemical concentration. Most strategies prefer to move in the upwind direction, with the exceptions being spiral, which favours downwind and upwind, and the *E. coli* algorithm, which never experienced this state. The third state, depicted in Figure 9c represents situations where the robot is sensing high odour concentrations. In this scenario, most strategies favour moving upwind. Similarly to State 2, the exceptions are spiral, which prefers to move downwind and upwind, and the *E. coli* algorithm, which never experienced this state. State 4 represents situations where the robot has recently lost contact with the chemical substance. In these situations, many approaches prefer to move in the upwind direction. The exceptions are the two approaches proposed by Grasso et al. which favour moving downwind, MP which gives preference to crosswind and SP which prefers both upwind and downwind. Moreover, the *E. coli* algorithm never perceived this state and its anemotactic variant slightly favours moving crosswind. The histograms from which these conclusions are drawn are plotted in Figure 9d. State 5 corresponds to situations where the robot has already sensed odour, but has lost contact with the chemical plume for an extended period of time. The histograms plotted in Figure 9e show that most strategies favour moving in the crosswind direction in this situation. The exceptions are G1, G2 and SP which prefer to move both in the downwind and upwind directions. Moreover, the *E. coli* algorithm has never experienced this state and its anemotactic variant does not seem to exhibit a clear preference in the direction of motion.

Similarly to environment 1, most strategies spent the majority of time in the first state, searching for the chemical plume. The exceptions are the strategies proposed by Grasso et al., which spend similar amounts of time trying to find the chemical plume (State 1) or attempting to re-encounter it (states 4 and 5). The remaining states have been experienced for significantly less amounts of time. This serves as an indication for the reduced chemical dispersion in this environment, making the plume searching stage a much harder, and thus, time consuming task.

#### 5.2.2. Statistical Analysis

This section reports the statistical analysis made to assess the differences between the behaviors of the strategies when operating in each state of Environment 2. Similarly to what was done for the advection-dominated environment, a preprocessing stage is made to ensure that all strategies have the same amount of state-action samples for each state. During this preprocessing, the strategies that have a low amount of samples in any state are removed from the analysis. The two variants of the *E. coli* algorithm were discarded, and the resulting sets have between 174 samples (state 3) and 56,255 samples (state 1).

The following step of the analysis consists of assessing whether the data follows normal distributions. Once again, the Kolmogorov–Smirnov test is applied, showing that at the chosen 95% confidence interval, the data of all strategies follow normal distributions. The next step consists of applying a parametric test to assess whether there are statistically significant differences between the behaviors exhibited by the strategies in each state. Similarly to what was done in Environment 1, the test chosen is the Independent One-Way Anova. The results of this test indicate that, at a 95% confidence interval, there are statistically significant differences between the behaviors of the strategies in all states, as all *p*-values are equal to 0, or may orders of magnitude lower than the significance value.

The last step of this analysis consists of using the Independent *t*-test to perform pairwise comparisons of the behaviours of the various strategies in each state. The results of this test are presented in Table 8. The significance value is again adjusted using the Bonferroni Correction, being the resulting value equal to 0.00089. In state 1, i.e., when the robot has not yet sensed odour, there are a total of 17 significant differences between the behaviours of the various strategies. The most consensual behaviours are those of MP, SM, SA, CT and DB, each having 4 statistically significant differences. The most disruptive behaviour is exhibited by SP, being significantly different from all other strategies. In state 2, i.e., when the robot is sensing a low amount of odour, there are 13 statistically significant differences out of the 28 comparisons made. The most consensual strategies are G1, SA and CT, each only being significantly different three strategies. The most disruptive behaviour is that of SP, being significantly different from 6 other strategies. When the robot is sensing high odour concentrations, state 3, there are 14 statistically significant differences out of the 28 comparisons made. The most consensual behaviours are exhibited by MP, G2 and SA each having 2 significant differences. The most disruptive behaviour is exhibited by SP being significantly different from all other strategies. State 4, i.e., when the robot has recently stopped sensing odour, is the state where the behaviours of the strategies are most dissimilar. Here, the only comparisons that are not significantly different are CT and DB; SM and DB; SM and CT; and G1 and G2. In state 5, i.e., when the robot has stopped sensing odour for a reasonable amount of time, there are 9 statistically significant differences between the behaviours of the various strategies, making it the most consensual state of this environment. In this state, the most disruptive behaviours are exhibited by G2, MP and SM, each being significantly different from 5 other strategies. The most consensual behaviour is that of G1, not being significantly different from any other strategy.

### 5.3. General State-Action Mappings

This section uses the state-action mappings produced by all strategies to draw conclusions about the most consensual actions to take in each state. In order to do so, for each environment, the data of all strategies was merged into a single set. The clusters were found, as in Section 5.1.1 and Section 5.2.1, and the corresponding histograms of relative frequency of actions were built. As in the previous sections, the actions of the search agents are characterized by the DCTU feature (Section 3.3.1) but, for more clarity, they are referred to as moving downwind, crosswind or upwind.

#### 5.3.1. Environment 1: Advection-Dominated

**Figure 10:**
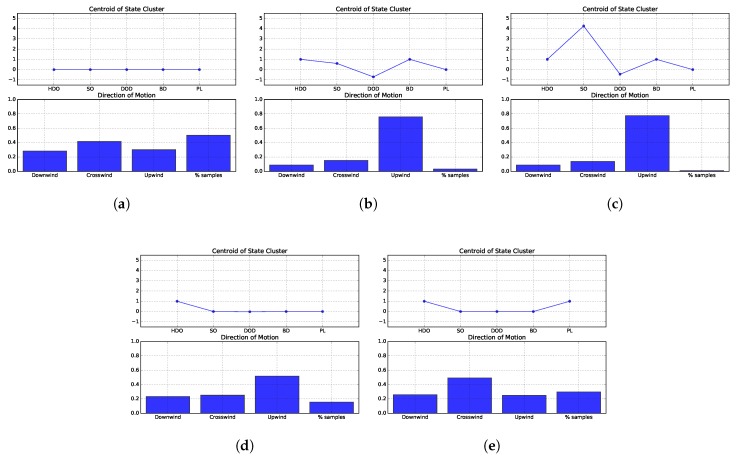
5 state-action mappings for environment 1. The top of each sub-figure presents the centroid of the respective state cluster. The bottom of each sub-figure presents the histograms of relative frequency of the actions taken by all strategies in the respective state, characterized by the direction of motion. A fourth column is presented in these histograms, representing the relative amount of simulation steps that all strategies spent perceiving the respective state. (**a**) State 1: the robot has yet to detect odour; (**b**) State 2: the robot is detecting low concentrations of odour; (**c**) State 3: the robot is detecting high concentrations of odour; (**d**) State 4: the robot has recently lost the chemical plume; (**e**) State 5: the robot has lost the odour plume for an extended time period.

The clusters of states for this environment were created using the same methods and parameters as in Section 5.1.1. State 1, Figure 10a, represents the situations where the robot has yet to sense odour. In this state the most consensual action is moving crosswind, having over 40% of relative frequency. The studied strategies spend the majority of the time in this state, using approximately 50% of the available simulation steps. The second state, Figure 10b, represents the situations where the robot is sensing a relatively low amount of chemical concentration. In this state the most consensual action is moving upwind. The studied strategies spend relatively small amounts of time perceiving this state, equating to less than 10% of the available simulation steps. The third state, Figure 10c, represents the situations where the robot is sensing high odour concentrations. This state is perceived for the shortest amount of time steps and, similarly to state 2, the most consensual action is, by far, moving upwind. The fourth state, Figure 10d, represents the situations where the robot has recently lost contact with the chemical plume. This state has been perceived for almost 20% of the available time, making it the third most commonly experienced state. The most consensual action in this state is moving upwind. Finally, state 5, Figure 10e, encompasses the situations where the robot has lost the odour plume for an extended time period. This state is perceived for approximately 30% of the available time and the most consensual action is moving crosswind.

#### 5.3.2. Environment 2: Diffusion-Dominated

**Figure 11:**
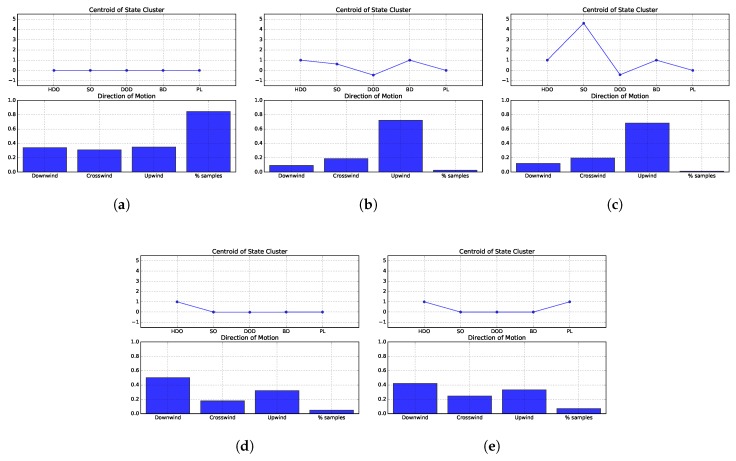
5 state-action mappings for environment 2. The top of each sub-figure presents the centroid of the respective state cluster. The bottom of each sub-figure presents the histograms of relative frequency of the actions taken by all strategies in the respective state, characterized by the direction of motion. A fourth column is presented in these histograms, representing the relative amount of simulation steps that all strategies spent perceiving the respective state. (**a**) State 1: the robot has yet to detect odour; (**b**) State 2: the robot is detecting low concentrations of odour; (**c**) State 3: the robot is detecting high concentrations of odour; (**d**) State 4: the robot has recently lost the odour plume; (**e**) State 5: the robot has lost the plume for an extended time period.

This section collectively analyses the state-action mappings produced by all strategies in Environment 2, i.e., an environment with a weak and unstable air flow. The methods and parameters used in Section 5.2.1 are also used here. State 1, depicted in Figure 11a, refers to situations where the robot must explore the environment, searching for the initial odour cues. In this environment the odour does not travel far from the source and thus, it is much harder to find the first chemical cue. This claim is supported by the fact that the robots spend over 80% of the available time perceiving state 1, which is almost twice the time spent in environment 1. There seems no be no clear consensus regarding the direction of motion in this state, with crosswind being slightly less preferred than the other directions. The second state, Figure 11b, represents the situations where the robot is sensing low chemical concentrations. Given the dispersion patterns created in this environment, the robot spends little time sensing odour, and, in particular, this state is perceived for less than 10% of the available time. Nevertheless, the most consensual action is moving upwind. The third state, Figure 11c, corresponds to situations where the robot is sensing high odour concentrations. This state is perceived for the least amount of time and, similarly to state 2, the most favoured direction of motion is upwind. The fourth state, Figure 11d, represents the situations where the robot has recently stopped sensing odour. In it, the most consensual action is moving downwind, which is chosen approximately 50% of the time, followed by moving upwind. The fifth state, Figure 11e, represents the situations where the robot has lost contact with odour for an extended time period. This is the second most perceived state and the most consensual direction of motion is downwind, followed by upwind.

## 6. Discussion

The previous section compared the state-action mappings produced by a range of search strategies in: (1) an advection-dominated environment and (2) a diffusion-dominated environment. For each environment, the perceptual states experienced by the robot were grouped into a reduced number of meaningful clusters and histograms of the actions taken were built for each cluster. A small set of rules can be derived from the results obtained:

1. If the robot has never detected odour and:
  (a) The odour dispersion is dominated by advection, then move crosswind;
  (b) The odour dispersion is dominated by diffusion, then move in any direction with similar probability;
2. If the robot senses odour, move upwind;
3. If the robot has recently lost contact with the chemical plume and:
  (a) The odour dispersion is dominated by advection, then move upwind;
  (b) The odour dispersion is dominated by diffusion, then move downwind;
4. If the robot has lost contact with the chemical plume for a long time period and:
  (a) The odour dispersion is dominated by advection, then move crosswind;
  (b) The odour dispersion is dominated by diffusion, then move downwind;

These rules indicate that, in the majority of states, the most preferred directions of motion are different for each environment. The exceptions are the second and third states, i.e. when the robots are sensing odour. In these situations, half of the considered strategies are designed to surge upwind, thus, biasing the histograms of actions. However, looking back to Figure 10b,c and Figure 11b,c, it is visible that the difference between the frequencies of moving upwind and moving in the other directions is very high, not being likely to be caused solely by the strategies that are designed to move upwind. Instead, it seems that the other strategies (e.g., the gradient-based approaches) are also moving upwind when sensing odour. This claim is supported by the fact that all strategies spend similar amounts of time perceiving these states, as can be seen from Figure 7b,c and Figure 9b,c.

The Independent *t*-test found that, in the advection-dominated environment, the strategies exhibit the most consensual behaviours prior to detecting any odour (state 1). In this state and environment, the most favoured action was moving crosswind, which is the default behaviour for 6 strategies. Considering that this environment contains a chemical plume extended along the wind direction, moving crosswind is likely to make the robot encounter odour. Conversely, in the diffusion-dominated environment, moving crosswind was the least favoured action, existing no clear distinction between downwind and upwind. While the bias created by the search strategies rendered the results of the advection-dominated environment non-surprising, the same can not be said for the second environment. The lack of a clear preference of motion in the absence of a strong air flow may be due to the various strategies perceiving each state for distinct time periods, and thus having different contributions for the histograms. As a result, even though 60% of the strategies are wired to move crosswind, the disparity in the times spent perceiving the first state in the diffusion-dominated environment create the conditions for not existing any consensual action. This result also indicates that, when the air-flow is weak and very unstable, the search for the initial odour detection should not be done based on the direction of the wind. This claim is supported by the fact that the three strategies that spend the least amount of time perceiving this state use no wind information to guide their search (Figure 9a). The Independent *t*-test also found that in the diffusion-dominated environment, the strategies exhibit the most consensual behaviours after having having lost the plume for extended time periods (state 5). The most consensual action for this state in the advection-dominated environment was moving crosswind. Due to the characteristics of the odour plume in this environment, moving in this direction is likely to make the robot re-encounter odour. Conversely, in the diffusion-dominated environment, the most consensual action was moving downwind. In this environment, the odour is clustered around the location of the source. Considering that the most consensual action is moving upwind when detecting odour, it is likely that state 5 is perceived due to the robot moving upwind past the location of the source. In such scenario, moving downwind should make the robot re-encounter odour.

Another result of the Independent *t*-test shows that, in both environments, the strategies exhibited the most disparate behaviours when perceiving the fourth state, i.e., whenever the robot has recently lost contact with the chemical substance. In the advection-dominated environment, the most preferred action for this state was moving upwind, whereas in the diffusion-dominated environment, the most favoured action was moving downwind. These different behaviours provide hints as to where the states are typically perceived. In the advection-dominated environment, the intermittent characteristics of the plume often causes the robot to transition between sensing odour (states 2 and 3) and loosing the plume (state 4). In such case, it is likely that the source is still upwind from the robot’s location, and thus, the strategies that move in that direction should perform better. Conversely, in the diffusion-dominated environment, the odour is clustered around the chemical source, existing no concentration voids in that region. As a result, if the robot stops sensing odour, it is likely to have gone past the location of the chemical source. Thus, as the robot starts the search process downwind from the chemical source and the most consensual action is moving upwind when detecting odour, moving downwind should be the most successful action for re-encountering odour.

Regarding the time used during the experiments, in the advection-dominated environment, the strategies spent the most time in states 1 and 5 (i.e., either before sensing any odour or having lost contact with it for a long time), representing over 70% of the available simulation steps. In the diffusion- dominated environment, the strategies spent more than 80% of the available time in state 1 (i.e., before having detected any odour). This provides a clear indication to the increased difficulty of locating the first chemical cue in the diffusion-dominated environment, especially considering that the arena is much smaller than the one used in the advection-dominated environment.

## 7. Conclusions and Future Work

This work reviewed, implemented and tested a set of reactive strategies for locating odour sources. Most of these strategies are bio-inspired, whereas others are human-designed, but show close similarities to bio-inspired ones. The strategies were tested in two distinct environments, differing in size and in air flow conditions, which influence the odour dispersion. In order to perform these tests, a purpose-built robotic simulator was devised. This simulator achieves a speed-up of over 1700 times real time, whilst retaining sufficient details to ensure the validity of the experiments. This speed-up makes the developed simulator adequate for learning and evolutionary robotics experiments, which typically require evaluating many candidate solutions. The data generated during simulations is kept in xml-inspired data structures supporting posterior analysis and reuse of experiments.

The strategies were compared from the state-action perspective, using the information contained in the generated datasets. The states perceived by the strategies were grouped into meaningful clusters and, for each cluster, histograms of actions were built. From these state-action mappings, it was possible to draw conclusions regarding the overall most common actions for each state of each environment: (1) If the robot has never sensed odour and (a) the odour dispersion is dominated by advection, then move crosswind; otherwise, (b) if the odour dispersion is dominated by diffusion, then move in any direction with similar probabilities. (2) If the robot is sensing odour, then move upwind regardless of the environmental conditions. (3) If the robot has recently lost contact with the chemical plume and (a) the odour dispersion is dominated by advection, then move upwind; otherwise, (b) if the dispersion is dominated by diffusion then move downwind. (4) If the robot has lost contact with the chemical plume for a long time period, and (a) the odour dispersion is dominated by advection, then move crosswind; otherwise, (b) if the odour dispersion is dominated by diffusion, then move downwind.

Statistical hypothesis were tested to assess which strategies behaved significantly different in each perceptual state of the two environments. From these tests it is possible to say that there are significant differences between the various strategies in all perceptual states. The fourth state of the advection-dominated environment (i.e., when the robot has recently lost contact with the chemical plume), is where the strategies have the most different behaviours, with a total of 35 significantly different comparisons out of the 36 made. Conversely, the fifth state of the diffusion-dominated environment (i.e., when the robot has lost contact with the chemical plume for a long time period) is where the strategies exhibit the most similar behaviours, with a total of nine significantly different comparisons out of the 28 made. Moreover, some strategies can be considered to behave similarly in a given state, but exhibit significantly different behaviours in others.

In the future, the conducted analysis should be extended to assess the performance of the various strategies. Those works should study a wider range of airflow conditions, as well as more complex environments, containing obstacles and multiple odour sources. The created datasets should be posteriorly used for training robotic controllers, which should result in faster training processes than performing complete simulations.

## Figures and Tables

**Table 1:** Simulator parameters.

Category	Parameters	Env. 1	Env. 2
Arena	Width (*w*)	70 m	35 m
Arena	Length (*l*)	70 m	35 m
Simulation	Step	0.5 seconds	
Simulation	Duration	600 seconds	
Wind	Grid Spacing	15% of the arena’s width	
Wind	Initial Velocity	(0.5, 0) m/s	(0.01, 0) m/s
Wind	Angular Variance	0.08 radians	0.5 radians
Odour	Chemical Emission Rate ($\bar{Q}$)	$8.3\cdot 10^{9}$ molecules/s	
Odour	Filament Emission Rate (*n*)	0.2 filaments/s	
Odour	Region of the Odour Source	(x,y)∼(w·U(0.2,0.4), l·U(0.25,0.75))	
Robot	Start Region	(x,y)∼(N(w−7,1.5),N(l−7,1.5))	
*# U and N respectively stand for uniform and normal distributions*			

**Table 2:** Structure of the behavioural dataset.

<step s>	# *s* is the index of the simulation step
<robot r>	# *r* is the robot id
<odometry>	# pose of the robot in global coordinates
<x x>	
<y y>	
<h h>	# heading of the robot in the global coordinate system
</odometry>	
<odour>	
<sensed_odour c>	# current gas concentration sensed
<best_odour $B_{c}>$	# highest gas concentration sensed
<best_odour_step $S_{Bc}>$	# simulation step when it was sensed
<best_odour_location>	# global coordinates of the highest gas concentration sensed
<x x>	
<y y>	
</best_odour_location>	
<lastOdourSensed_concentration $L_{c}>$	# concentration of the most recent detection
<lastOdourSensed_step $S_{Lc}>$	# simulation step when it was sensed
<last_odour_location>	# global coordinates of the most recent odour detection
<x x>	
<y y>	
</last_odour_location>	
<plume_lost P>	# binary variable informing if the plume has been lost for a long time
</odour>	
<wind>	
<wind_speed $w_{s}>$	# current wind speed
<upwind_dir u_dir>	# upwind angle in the robot’s coordinate system
<downwind_dir d_dir>	# downwind angle in the robot’s coordinate system
<crosswind_dir x_dir>	# crosswind angle in the robot’s coordinate system
</wind>	
<scan>	
<n_readings n>	# number of laser beams
<fov f>	# field of view of the laser range finder (LRF)
<start_angle $s_{\alpha }>$	# orientation of the first beam
<angle_increment $inc_{\alpha }>$	# angle between consecutive beams
<max_range R>	# maximum LRF range
<readings [$r_{0},r_{1},\ldots ,r_{n}$]>	
</scan>	
<target>	# motion vector of the robot in its coordinate system
<x x>	
<y y>	
</target>	
<behaviour>	
<B>	# id of the atomic behaviour performed (e.g., move, rotate, etc)
</behaviour>	
</robot r>	
</step s>	

**Table 3:** Designed features.

Feature	Values	Description
*Plume*_*lost* (PL)	{0,1}	A feature that takes the value 1 if the robot has detected odour in the past, but has not sensed it for an extended period of time, i.e., a time period over *plume_lost_threshold* (Table 4). Otherwise, its value is 0.
*Binary*_*odour*_*detection* (BD)	{0,1}	A feature that informs whether the robot is detecting odour (1) or not (0).
*Discrete*_*odour*_*difference* (DOD)	{−1,0,1}	A feature that informs whether the chemical concentration being detected by the robot is higher than in the previous step (1), equal to the previous step (0) or lower than in the previous step (−1)
*Has*_*detected*_*odour* (HDO)	{0,1}	A feature that informs whether the robot has ever detected odour (1) or not (0).
*Discrete*_*cos*_*target*_*upwind* (DCTU)	{−1,0,1}	A feature that computes the cosine of the robot’s goal vector in relation to the upwind direction. It then divides the range of the cosine values into 3 equal intervals and yields −1, 0 or 1 if the robot’s motion is more aligned with the downwind, crosswind or upwind direction.

**Table 4:** Parameters used in the various strategies.

Parameter	Value	Algorithm
$\theta^{\prime }$	π/12 radians	EC, AE, G1, G2
$\theta^{\prime \prime }$	π/3 radians	DB
θ	π/4 radians	SP, SM, MP, SA, CT
Θ	π radians	EC, AE
*l*	1 m	EC, AE, G1, G2
*L*	2 m	EC, AE, SP, SM, MP, SA, CT, DB
$T_{1}$	0 molecules/cm${}^{3}$	G1, G2
$T_{2}$	0 molecules/cm${}^{3}$	G2
$K_{\mu }$	0.5	SP
$K_{P}$	0.5	SP
$mT_{PI}$	0.5	SP
*plume*_*lost*_*threshold*	50 s	DB, SM, MP, CT, SA
*odour*_*tracking*_*threshold*	$2.5\times 10^{6}$ molecules/cm${}^{3}$	MP

**Table 5:** Parameters used for validating the simulator.

Category	Parameter	Value
Arena	Width (*w*)	100 m
Arena	Length (*l*)	100 m
Wind	Grid Spacing	7 m
Wind	Initial Velocity	(1.0, 0.0) m/s
Wind	Angular Variance	0.02 radians
Wind	Kx	10
Odour	Filament Emission Rate (*n*)	10 filaments/s
Odour	Initial Filament Radius (*R*)	0.03162 m
Odour	Filament growth rate (γ)	0.001 m${}^{2}$/s
Odour	Source Position	(5,50) m
Simulation	Step	0.01 seconds
Simulation	Duration	seconds

**Table 6:** Statistics of the odour plumes created in the real-world by Jones, modelled by Farrell et al. and simulated by the simulator proposed in this paper. The data is collected using a 1 Hz filter bandwidth. It is presented in three lines, respectively corresponding to the sensors at 2 m, 5 m and 10 m downwind from the chemical source.

		Jones	Proposed Simulator	Farrell et al.
2 m	Skewness	4.18	1.13	2.32
	Kurtosis	23.2	1.48	2.89
	Peak to mean ratio	13.9	5.63	11.76
	Intermittency	79.1	26.5	28.11
	std dev./mean	0.90	1.14	1.73
5 m	Skewness	4.24	3.27	3.80
	Kurtosis	23.7	4.17	5.08
	Peak to mean ratio	22.2	17.39	23.58
	Intermittency	81.0	70.68	47.56
	std dev./mean	1.96	2.35	2.45
10 m	Skewness	4.49	5.29	5.02
	Kurtosis	30.6	7.12	7.21
	Peak to mean ratio	28.5	30.78	46.86
	Intermittency	83.7	83.08	59.54
	std dev./mean	1.65	3.28	2.94

**Table 7:** Independent *t*-test applied to the data obtained from Environment 1. The Bonferroni correction was used to adjust the significance value, so that, at a confidence level of 95%, two strategies are significantly different if their *p* value is lower than 0.00069. The *p* values of such comparisons are typeset in boldface.

Algorithms		State 1		State 2		State 3		State 4		State 5	
AE	G1	st≈−18.171	***p*** ≈ **0.0**	st≈−18.478	***p*** ≈ **0.0**	st≈−8.209	***p*** ≈ **0.0**	st≈−21.236	***p*** ≈ **0.0**	st≈9.334	***p*** ≈ **0.0**
AE	G2	st≈−19.98	***p*** ≈ **0.0**	st≈−0.241	p≈0.80969	st≈0.951	p≈0.34203	st≈22.672	***p*** ≈ **0.0**	st≈−7.772	***p*** ≈ **0.0**
AE	DB	st≈0.062	p≈0.95083	st≈−11.695	***p*** ≈ **0.0**	st≈−1.72	p≈0.08578	st≈−63.663	***p*** ≈ **0.0**	st≈3.192	p≈0.00141
AE	SA	st≈−6.995	***p*** ≈ **0.0**	st≈−23.856	***p*** ≈ **0.0**	st≈−9.882	***p*** ≈ **0.0**	st≈−92.063	***p*** ≈ **0.0**	st≈4.526	***p*** ≈ **1 × 10^−5^**
AE	MP	st≈0.062	p≈0.95083	st≈−20.904	***p*** ≈ **0.0**	st≈−11.596	***p*** ≈ **0.0**	st≈−0.266	p≈0.79046	st≈−1.338	p≈0.18098
AE	SP	st≈−2.041	p≈0.04129	st≈6.127	***p*** ≈ **0.0**	st≈5.636	***p*** ≈ **0.0**	st≈9.084	***p*** ≈ **0.0**	st≈−3.927	***p*** ≈ **9 × 10^−5^**
AE	SM	st≈0.062	p≈0.95083	st≈−27.268	***p*** ≈ **0.0**	st≈−12.653	***p*** ≈ **0.0**	st≈−45.146	***p*** ≈ **0.0**	st≈6.891	***p*** ≈ **0.0**
AE	CT	st≈−6.361	***p*** ≈ **0.0**	st≈−15.31	***p*** ≈ **0.0**	st≈−3.962	***p*** ≈ **8 × 10^−5^**	st≈−28.768	***p*** ≈ **0.0**	st≈4.544	***p*** ≈ **1 × 10^−5^**
G1	G2	st≈−1.574	p≈0.11545	st≈17.346	***p*** ≈ **0.0**	st≈8.714	***p*** ≈ **0.0**	st≈41.001	***p*** ≈ **0.0**	st≈−15.543	***p*** ≈ **0.0**
G1	DB	st≈21.392	***p*** ≈ **0.0**	st≈5.89	***p*** ≈ **0.0**	st≈5.985	***p*** ≈ **0.0**	st≈−33.717	***p*** ≈ **0.0**	st≈−8.109	***p*** ≈ **0.0**
G1	SA	st≈13.924	***p*** ≈ **0.0**	st≈−5.735	***p*** ≈ **0.0**	st≈−1.748	p≈0.08071	st≈−53.212	***p*** ≈ **0.0**	st≈−6.118	***p*** ≈ **0.0**
G1	MP	st≈21.392	***p*** ≈ **0.0**	st≈−2.526	p≈0.0116	st≈−3.954	***p*** ≈ **8 × 10^−5^**	st≈22.79	***p*** ≈ **0.0**	st≈−11.959	***p*** ≈ **0.0**
G1	SP	st≈14.868	***p*** ≈ **0.0**	st≈23.843	***p*** ≈ **0.0**	st≈13.387	***p*** ≈ **0.0**	st≈28.065	***p*** ≈ **0.0**	st≈−12.091	***p*** ≈ **0.0**
G1	SM	st≈21.392	***p*** ≈ **0.0**	st≈−10.046	***p*** ≈ **0.0**	st≈−5.258	***p*** ≈ **0.0**	st≈−19.161	***p*** ≈ **0.0**	st≈−4.514	***p*** ≈ **1 × 10^−5^**
G1	CT	st≈14.542	***p*** ≈ **0.0**	st≈3.007	p≈0.00266	st≈4.017	***p*** ≈ **6 × 10^−5^**	st≈−5.291	***p*** ≈ **0.0**	st≈−5.786	***p*** ≈ **0.0**
G2	DB	st≈23.558	***p*** ≈ **0.0**	st≈−11.008	***p*** ≈ **0.0**	st≈−2.568	p≈0.01038	st≈−83.937	***p*** ≈ **0.0**	st≈11.688	***p*** ≈ **0.0**
G2	SA	st≈15.905	***p*** ≈ **0.0**	st≈−22.308	***p*** ≈ **0.0**	st≈−10.244	***p*** ≈ **0.0**	st≈−111.758	***p*** ≈ **0.0**	st≈12.293	***p*** ≈ **0.0**
G2	MP	st≈23.558	***p*** ≈ **0.0**	st≈−19.596	***p*** ≈ **0.0**	st≈−11.806	***p*** ≈ **0.0**	st≈−24.991	***p*** ≈ **0.0**	st≈7.863	***p*** ≈ **0.0**
G2	SP	st≈16.513	***p*** ≈ **0.0**	st≈6.178	***p*** ≈ **0.0**	st≈4.546	***p*** ≈ **1 × 10^−5^**	st≈−12.281	***p*** ≈ **0.0**	st≈3.542	***p*** ≈ **0.0004**
G2	SM	st≈23.558	***p*** ≈ **0.0**	st≈−25.454	***p*** ≈ **0.0**	st≈−12.751	***p*** ≈ **0.0**	st≈−65.554	***p*** ≈ **0.0**	st≈14.58	***p*** ≈ **0.0**
G2	CT	st≈16.533	***p*** ≈ **0.0**	st≈−14.393	***p*** ≈ **0.0**	st≈−4.726	***p*** ≈ **0.0**	st≈−49.455	***p*** ≈ **0.0**	st≈12.113	***p*** ≈ **0.0**
DB	SA	st≈−9.41	***p*** ≈ **0.0**	st≈−10.948	***p*** ≈ **0.0**	st≈−7.502	***p*** ≈ **0.0**	st≈−21.228	***p*** ≈ **0.0**	st≈2.092	p≈0.03642
DB	MP	st≈0.0	p≈1.0	st≈−8.165	***p*** ≈ **0.0**	st≈−9.123	***p*** ≈ **0.0**	st≈71.854	***p*** ≈ **0.0**	st≈−6.258	***p*** ≈ **0.0**
DB	SP	st≈−2.458	p≈0.01399	st≈17.258	***p*** ≈ **0.0**	st≈7.094	***p*** ≈ **0.0**	st≈66.99	***p*** ≈ **0.0**	st≈−7.281	***p*** ≈ **0.0**
DB	SM	st≈0.0	p≈1.0	st≈−14.408	***p*** ≈ **0.0**	st≈−10.082	***p*** ≈ **0.0**	st≈15.451	***p*** ≈ **0.0**	st≈5.112	***p*** ≈ **0.0**
DB	CT	st≈−8.592	***p*** ≈ **0.0**	st≈−3.026	p≈0.0025	st≈−2.083	p≈0.03748	st≈30.625	***p*** ≈ **0.0**	st≈2.2	p≈0.02782
SA	MP	st≈9.41	***p*** ≈ **0.0**	st≈3.245	p≈0.00119	st≈−2.406	p≈0.01631	st≈109.845	***p*** ≈ **0.0**	st≈−7.356	***p*** ≈ **0.0**
SA	SP	st≈3.914	***p*** ≈ **9 × 10^−5^**	st≈28.828	***p*** ≈ **0.0**	st≈14.905	***p*** ≈ **0.0**	st≈90.956	***p*** ≈ **0.0**	st≈−8.195	***p*** ≈ **0.0**
SA	SM	st≈9.41	***p*** ≈ **0.0**	st≈−4.86	***p*** ≈ **0.0**	st≈−3.821	***p*** ≈ **0.00014**	st≈36.741	***p*** ≈ **0.0**	st≈2.442	p≈0.01462
SA	CT	st≈0.781	p≈0.43461	st≈8.538	***p*** ≈ **0.0**	st≈5.634	***p*** ≈ **0.0**	st≈52.701	***p*** ≈ **0.0**	st≈0.204	p≈0.83874
MP	SP	st≈−2.458	p≈0.01399	st≈26.107	***p*** ≈ **0.0**	st≈16.366	***p*** ≈ **0.0**	st≈10.099	***p*** ≈ **0.0**	st≈−3.415	***p*** ≈ **0.00064**
MP	SM	st≈0.0	p≈1.0	st≈−7.785	***p*** ≈ **0.0**	st≈−1.324	p≈0.18595	st≈−49.904	***p*** ≈ **0.0**	st≈10.84	***p*** ≈ **0.0**
MP	CT	st≈−8.592	***p*** ≈ **0.0**	st≈5.465	***p*** ≈ **0.0**	st≈7.43	***p*** ≈ **0.0**	st≈−31.41	***p*** ≈ **0.0**	st≈7.161	***p*** ≈ **0.0**
SP	SM	st≈2.458	p≈0.01399	st≈−31.899	***p*** ≈ **0.0**	st≈−17.275	***p*** ≈ **0.0**	st≈−50.351	***p*** ≈ **0.0**	st≈10.353	***p*** ≈ **0.0**
SP	CT	st≈−3.343	p≈0.00083	st≈−20.824	***p*** ≈ **0.0**	st≈−9.335	***p*** ≈ **0.0**	st≈−35.313	***p*** ≈ **0.0**	st≈8.125	***p*** ≈ **0.0**
SM	CT	st≈−8.592	***p*** ≈ **0.0**	st≈12.497	***p*** ≈ **0.0**	st≈8.493	***p*** ≈ **0.0**	st≈14.846	***p*** ≈ **0.0**	st≈−2.101	p≈0.03567

**Table 8:** Independent *t*-test applied to the data obtained from Environment 2. The Bonferroni correction was used to adjust the significance value, so that, at a confidence level of 95%, two strategies are significantly different if their *p* value is lower than 0.00089. The *p* values of such comparisons are typeset in boldface.

Algorithms		State 1		State 2		State 3		State 4		State 5	
MP	G2	st≈−25.078	***p*** ≈ **0.0**	st≈3.681	***p*** ≈ **0.00026**	st≈1.756	p≈0.08003	st≈8.851	***p*** ≈ **0.0**	st≈6.337	***p*** ≈ **0.0**
MP	G1	st≈−25.691	***p*** ≈ **0.0**	st≈1.144	p≈0.25344	st≈2.917	p≈0.00376	st≈4.869	***p*** ≈ **0.0**	st≈2.442	p≈0.01473
MP	SM	st≈−0.199	p≈0.84216	st≈0.348	p≈0.72825	st≈−0.657	p≈0.51179	st≈−15.481	***p*** ≈ **0.0**	st≈6.593	***p*** ≈ **0.0**
MP	SA	st≈1.185	p≈0.23583	st≈2.051	p≈0.04093	st≈2.506	p≈0.01267	st≈−34.408	***p*** ≈ **0.0**	st≈1.147	p≈0.25166
MP	SP	st≈−7.009	***p*** ≈ **0.0**	st≈9.683	***p*** ≈ **0.0**	st≈12.093	***p*** ≈ **0.0**	st≈−3.769	***p*** ≈ **0.00017**	st≈1.526	p≈0.12709
MP	CT	st≈1.743	p≈0.08134	st≈0.997	p≈0.31946	st≈−1.046	p≈0.29608	st≈−12.308	***p*** ≈ **0.0**	st≈1.755	p≈0.07948
MP	DB	st≈0.33	p≈0.74167	st≈6.349	***p*** ≈ **0.0**	st≈5.57	***p*** ≈ **0.0**	st≈−13.07	***p*** ≈ **0.0**	st≈4.634	***p*** ≈ **0.0**
G2	G1	st≈−0.556	p≈0.57853	st≈−2.577	p≈0.01031	st≈1.141	p≈0.25472	st≈−3.239	p≈0.00123	st≈−3.201	p≈0.0014
G2	SM	st≈24.903	***p*** ≈ **0.0**	st≈−3.429	***p*** ≈ **0.00067**	st≈−2.363	p≈0.0187	st≈−22.523	***p*** ≈ **0.0**	st≈−0.678	p≈0.49786
G2	SA	st≈25.946	***p*** ≈ **0.0**	st≈−1.976	p≈0.04882	st≈0.614	p≈0.53949	st≈−39.966	***p*** ≈ **0.0**	st≈−5.239	***p*** ≈ **0.0**
G2	SP	st≈16.923	***p*** ≈ **0.0**	st≈5.809	***p*** ≈ **0.0**	st≈10.527	***p*** ≈ **0.0**	st≈−11.038	***p*** ≈ **0.0**	st≈−4.085	***p*** ≈ **5 × 10^−5^**
G2	CT	st≈26.47	***p*** ≈ **0.0**	st≈−2.841	p≈0.00472	st≈−2.595	p≈0.00988	st≈−19.452	***p*** ≈ **0.0**	st≈−4.791	***p*** ≈ **0.0**
G2	DB	st≈25.41	***p*** ≈ **0.0**	st≈2.623	p≈0.00904	st≈4.111	***p*** ≈ **5 × 10^−5^**	st≈−20.285	***p*** ≈ **0.0**	st≈−2.888	p≈0.00393
G1	SM	st≈25.516	***p*** ≈ **0.0**	st≈−0.833	p≈0.40515	st≈−3.512	***p*** ≈ **0.0005**	st≈−18.052	***p*** ≈ **0.0**	st≈2.894	p≈0.00385
G1	SA	st≈26.554	***p*** ≈ **0.0**	st≈0.786	p≈0.43225	st≈−0.583	p≈0.5604	st≈−32.864	***p*** ≈ **0.0**	st≈−1.48	p≈0.139
G1	SP	st≈17.491	***p*** ≈ **0.0**	st≈8.536	***p*** ≈ **0.0**	st≈9.537	***p*** ≈ **0.0**	st≈−7.568	***p*** ≈ **0.0**	st≈−0.813	p≈0.41606
G1	CT	st≈27.078	***p*** ≈ **0.0**	st≈−0.201	p≈0.84098	st≈−3.672	***p*** ≈ **0.00028**	st≈−15.277	***p*** ≈ **0.0**	st≈−1.023	p≈0.30648
G1	DB	st≈26.024	***p*** ≈ **0.0**	st≈5.243	***p*** ≈ **0.0**	st≈3.149	p≈0.00178	st≈−15.962	***p*** ≈ **0.0**	st≈1.003	p≈0.31596
SM	SA	st≈1.383	p≈0.16668	st≈1.73	p≈0.08431	st≈3.158	p≈0.00173	st≈−12.49	***p*** ≈ **0.0**	st≈−5.274	***p*** ≈ **0.0**
SM	SP	st≈−6.828	***p*** ≈ **0.0**	st≈9.48	***p*** ≈ **0.0**	st≈12.549	***p*** ≈ **0.0**	st≈9.391	***p*** ≈ **0.0**	st≈−3.881	***p*** ≈ **0.00011**
SM	CT	st≈1.941	p≈0.05229	st≈0.666	p≈0.50582	st≈−0.465	p≈0.6425	st≈2.494	p≈0.01275	st≈−4.76	***p*** ≈ **0.0**
SM	DB	st≈0.529	p≈0.59669	st≈6.126	***p*** ≈ **0.0**	st≈6.021	***p*** ≈ **0.0**	st≈2.155	p≈0.03137	st≈−2.552	p≈0.01081
SA	SP	st≈−8.044	***p*** ≈ **0.0**	st≈8.119	***p*** ≈ **0.0**	st≈10.239	***p*** ≈ **0.0**	st≈21.673	***p*** ≈ **0.0**	st≈0.568	p≈0.57026
SA	CT	st≈0.551	p≈0.58147	st≈−1.039	p≈0.29926	st≈−3.335	p≈0.00095	st≈14.988	***p*** ≈ **0.0**	st≈0.573	p≈0.56683
SA	DB	st≈−0.861	p≈0.3893	st≈4.745	***p*** ≈ **0.0**	st≈3.72	***p*** ≈ **0.00023**	st≈15.083	***p*** ≈ **0.0**	st≈3.225	p≈0.00128
SP	CT	st≈8.563	***p*** ≈ **0.0**	st≈−8.899	***p*** ≈ **0.0**	st≈−12.476	***p*** ≈ **0.0**	st≈−6.926	***p*** ≈ **0.0**	st≈−0.095	p≈0.92421
SP	DB	st≈7.322	***p*** ≈ **0.0**	st≈−3.137	p≈0.00183	st≈−5.858	***p*** ≈ **0.0**	st≈−7.418	***p*** ≈ **0.0**	st≈2.024	p≈0.04316
CT	DB	st≈−1.419	p≈0.1559	st≈5.552	***p*** ≈ **0.0**	st≈6.1	***p*** ≈ **0.0**	st≈−0.389	p≈0.69743	st≈2.637	p≈0.00845

## Abbreviations

The following abbreviations are used in this manuscript:

AE	Anemotactic *E. coli* strategy, Section 3.4.1
BD	*Binary*_*odour*_*detection* feature
CT	Casting and Counter-turning, Section 2.2.3
DB	Dung Beelte strategy, Section 2.2.2
DOD	*Discrete*_*odour*_*difference* feature
EC	*E. coli* chemotaxis, Section 2.1.1
G1	Grasso et al.’s first strategy, Section 2.1.2
G2	Grasso et al.’s second strategy, Section 2.1.2
HDO	*Has*_*detected*_*odour* feature
LRF	Laser Range Finder
MP	Ishida et al.’s Multiphase strategy, Section 2.2.4
PL	*Plume*_*lost* feature
SA	Casting and Surge-Anemotaxis, Section 2.2.3
SM	Modified Silkworm Moth strategy, Section 2.2.1
SO	*Sensed*_*odour* feature
SP	Spiral, Section 2.1.3
